# Correction: USP7 attenuates endoplasmic reticulum stress-induced apoptotic cell death through deubiquitination and stabilization of FBXO7

**DOI:** 10.1371/journal.pone.0297970

**Published:** 2024-01-24

**Authors:** Su Hyoun Lee, Kwang Chul Chung

## Notice of republication

This article was republished on January 17^th^, 2024, to correct errors in Figs 1, 3 and 4, and to include missing Supporting Information files S1 Data, S2 Data, and S3 Data. The underlying western blots were erroneously published in place of the intended figures and the intended Supporting Information files were not published.

In addition, during editorial follow up, the corresponding author stated that there was an error in the data preparation process for the raw image files for the α-tubulin panel in Fig 3B and the HSP90 panel in Fig 3C. The correct files have now been provided in S1 Data.

Please download this article again to view the correct version. The originally published, uncorrected article and the republished, corrected article are provided here for reference.

## Supporting information

S1 File Originally published, uncorrected article. (PDF)

S2 File Republished, corrected article. (PDF)
