# Supplementary material for: USP7 attenuates endoplasmic reticulum stress-induced apoptotic cell death through deubiquitination and stabilization of FBXO7
Source: PLoS One. 2023 Oct 24;18(10):e0290371. doi: 10.1371/journal.pone.0290371 (PMC10597484; doi:10.1371/journal.pone.0290371)
Supplement: S1 Fig — (PDF) [file pone.0290371.s001.pdf]

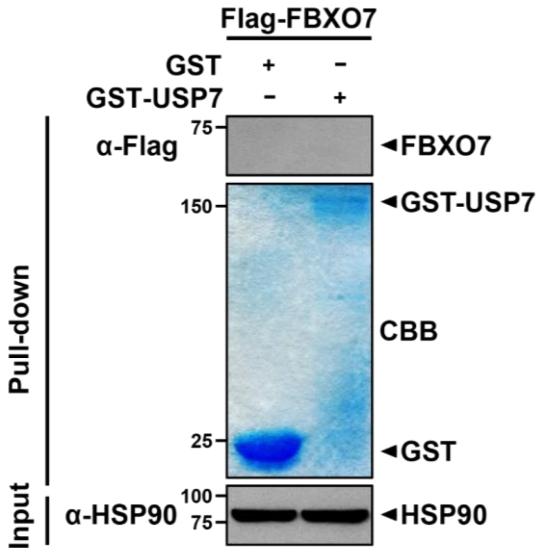

Figure S1. USP7 indirectly binds to FBXO7. Where specified, an in vitro GST pull-down assay was performed by incubating either bacterial recombinant GST-USP7 or GST protein (as a control) immobilized on glutathione-sepharose 4B beads with the lysates prepared from HEK293 cells transiently transfected with Flag-FBXO7. After the beads were washed with washing buffer, the bound proteins were eluted with elution buffer and analyzed by immunoblotting with anti-Flag antibody. The purity of GST-USP7 and GST proteins was identified by gel staining with Coomassie Brilliant Blue (CBB). HSP90 served as a loading control.
