# Supplementary material for: USP7 attenuates endoplasmic reticulum stress-induced apoptotic cell death through deubiquitination and stabilization of FBXO7
Source: PLoS One. 2023 Oct 24;18(10):e0290371. doi: 10.1371/journal.pone.0290371 (PMC10597484; doi:10.1371/journal.pone.0290371)
Supplement: S2 Fig — (PDF) [file pone.0290371.s002.pdf]

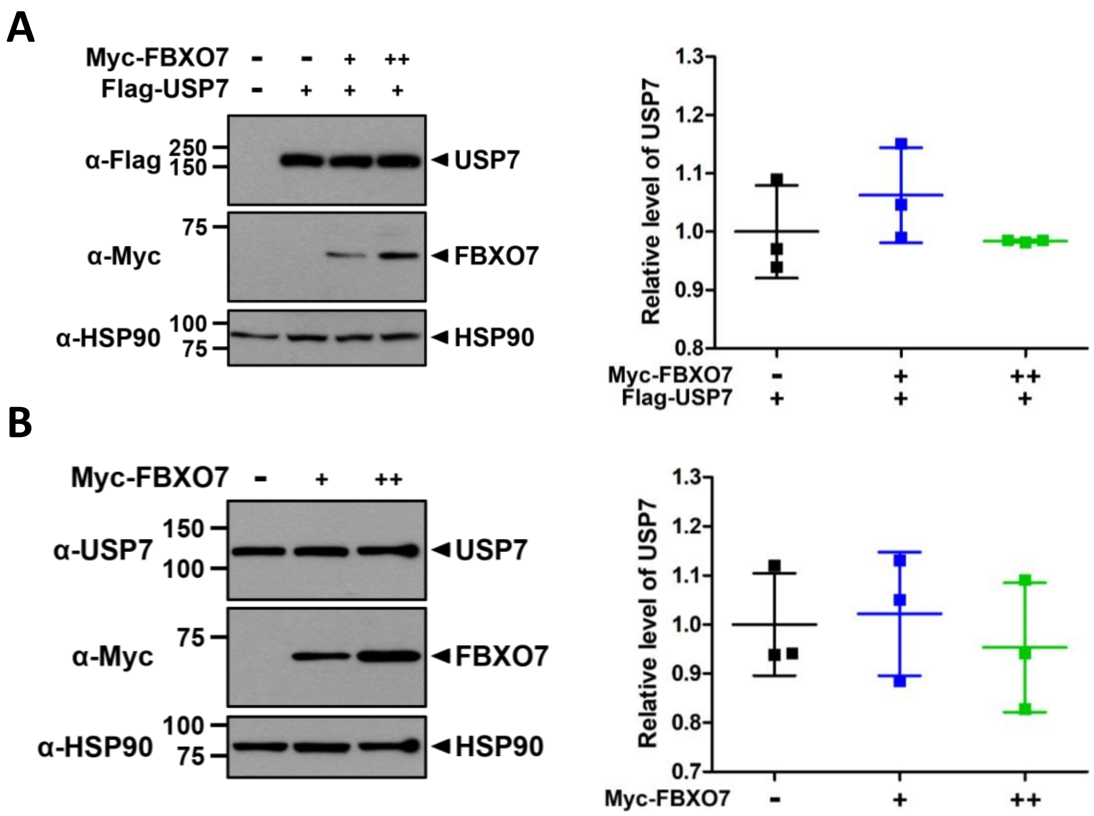

Figure S2. FBXO7 does not affect the USP7 level. ***A,*** Where specified, HEK293 cells were transfected for 24 h with a plasmid encoding wild type Flag-USP7 alone or together with increasing amounts of Myc-FBXO7. Cell lysates were immunoblotted with anti-Flag or anti-Myc antibodies. Relative USP7 levels were quantified, and the data are presented as the means ± SD of three independent experiments. ***B,*** HEK293 cells were transfected for 24 h with increasing amounts of plasmid encoding Myc-FBXO7. Cell lysates were immunoblotted with anti-USP7 or anti-Myc antibodies. Relative USP7 levels were quantified, and the data are presented as the means ± SD of three independent experiments. HSP90 served as a loading control.
