# Supplementary material for: USP7 attenuates endoplasmic reticulum stress-induced apoptotic cell death through deubiquitination and stabilization of FBXO7
Source: PLoS One. 2023 Oct 24;18(10):e0290371. doi: 10.1371/journal.pone.0290371 (PMC10597484; doi:10.1371/journal.pone.0290371)
Supplement: S3 Fig — (PDF) [file pone.0290371.s003.pdf]

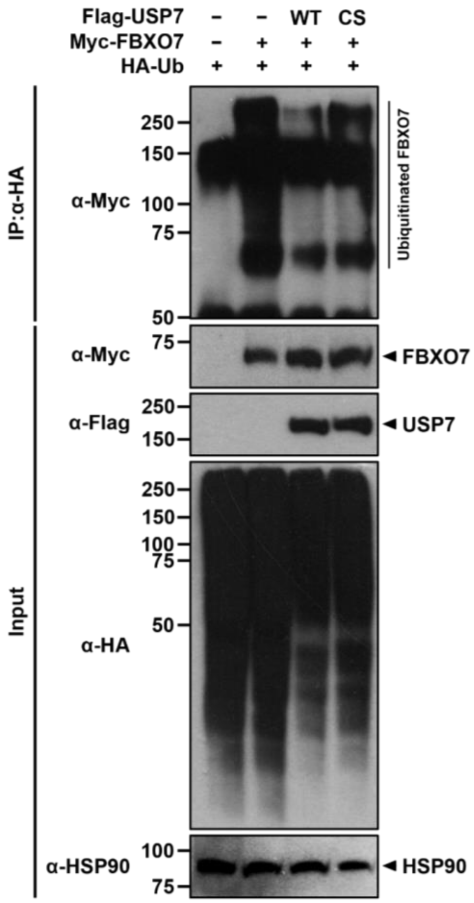

Figure S3. USP7 deubiquitinates the FBXO7. A, Where specified, HEK293 cells were transfected for 24 h with the plasmids encoding HA-Ubiquitin (Ub), Myc-FBXO7, Flag-USP7-WT, or Flag-USP7-C223S (CS) alone or in combination, and treated for 6 h with MG132 (20 μM). Cell lysates were immunoprecipitated with anti-HA antibody, followed by immunoblotting with the indicated antibodies. HSP90 served as a loading control.
