# Supplementary material for: USP7 attenuates endoplasmic reticulum stress-induced apoptotic cell death through deubiquitination and stabilization of FBXO7
Source: PLoS One. 2023 Oct 24;18(10):e0290371. doi: 10.1371/journal.pone.0290371 (PMC10597484; doi:10.1371/journal.pone.0290371)
Supplement: S4 Fig — (PDF) [file pone.0290371.s004.pdf]

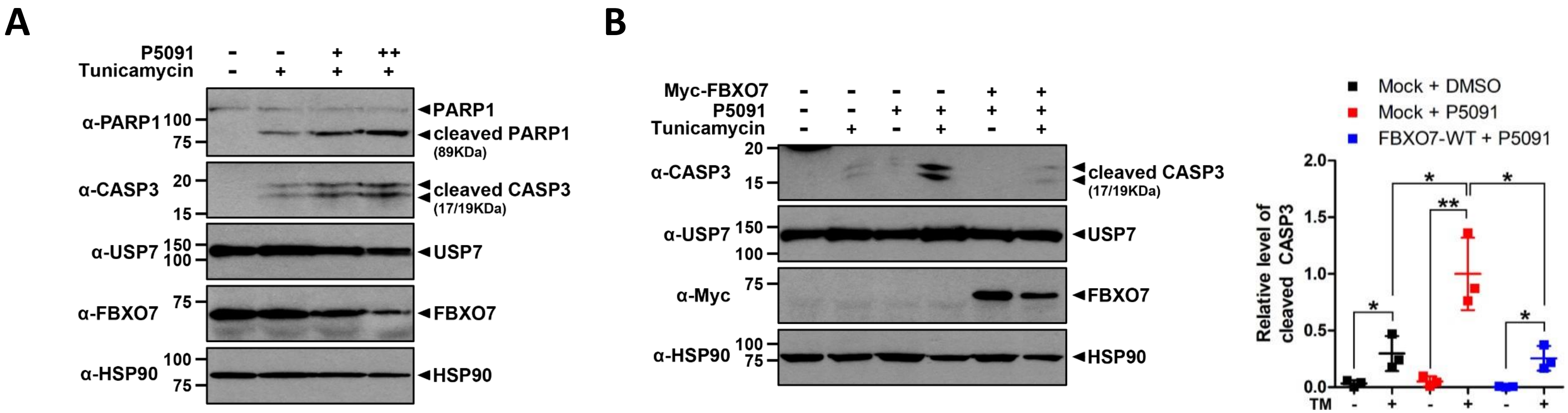

Figure S4. USP7 attenuates ER stress-induced apoptosis in SH-SY5Y cells. ***A,*** Where specified, SH-SY5Y cells were left untreated or treated for 24 h with TM (0.5 μM) or/and P5091 (5 or 10 μM). Cell lysates were immunoblotted with indicated antibodies. ***B,*** SH-SY5Y cells were transfected for 24 h with a plasmid encoding Myc-FBXO7, and treated for an additional 24 h with vehicle (-) or 0.5 μM TM alone or in combination with P5091 (10 μM). Cell lysates were immunoblotted with indicated antibodies. Data are presented as the mean ± SD of three independent experiments (**$p \leq 0.001$; * $p \leq 0.05$). HSP90 served as a loading control.
