# Supplementary figures and images for: USP7 attenuates endoplasmic reticulum stress-induced apoptotic cell death through deubiquitination and stabilization of FBXO7
Source: PLoS One. 2023 Oct 24;18(10):e0290371. doi: 10.1371/journal.pone.0290371 (PMC10597484; doi:10.1371/journal.pone.0290371)

**Figure S1.**

**pull down**

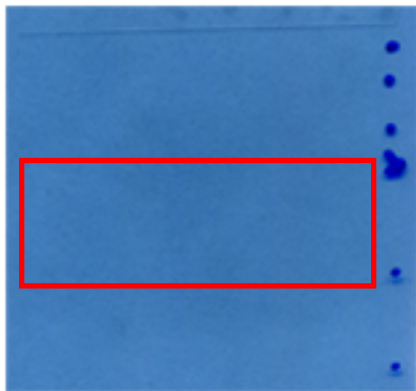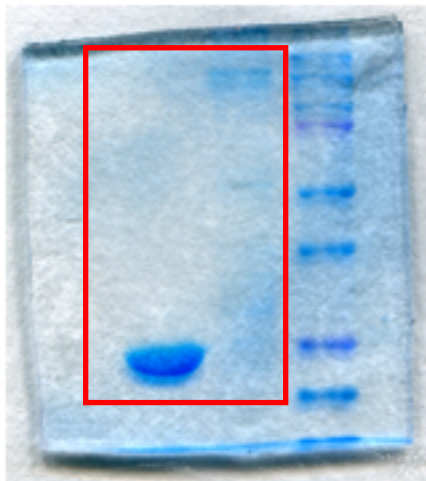

**input**

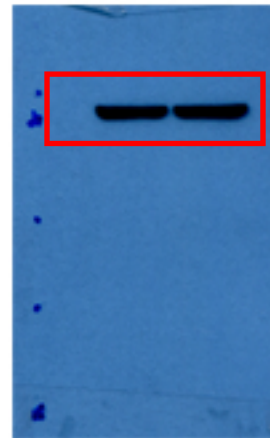

Supplement: S1 Raw image — (PDF) [file pone.0290371.s005.pdf]

Figure S2.

A

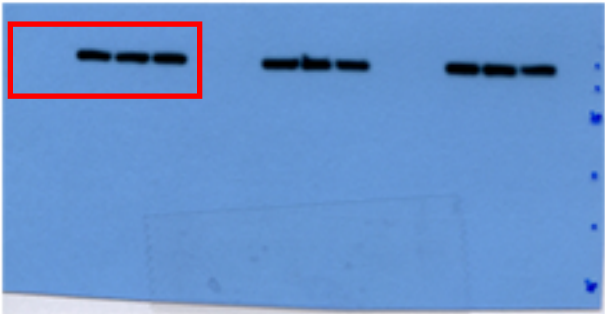

USP7

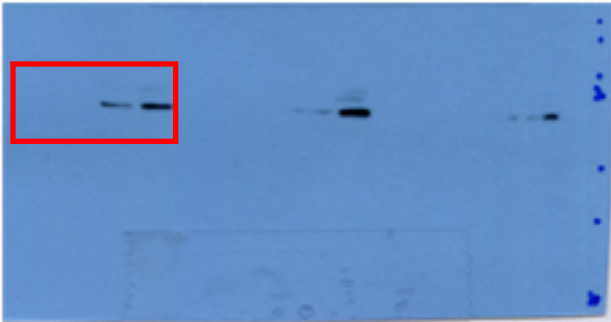

FBXO7

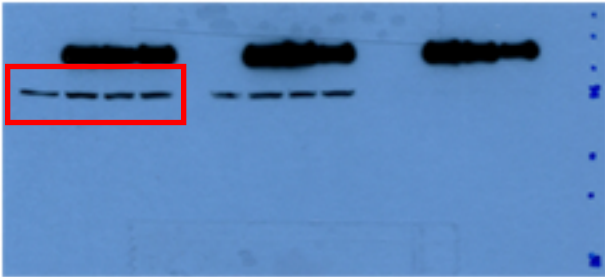

HSP90

B

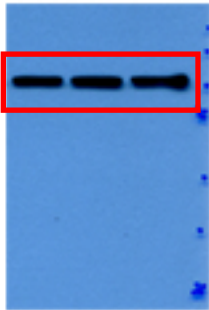

USP7

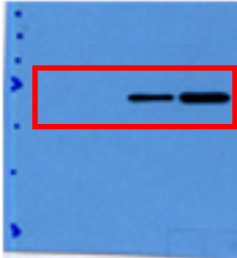

FBXO7

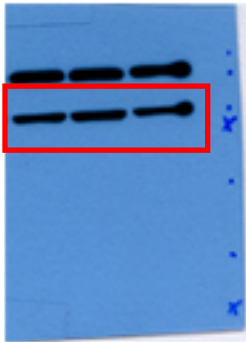

HSP90

Supplement: S2 Raw image — (PDF) [file pone.0290371.s006.pdf]

**Figure S3.**

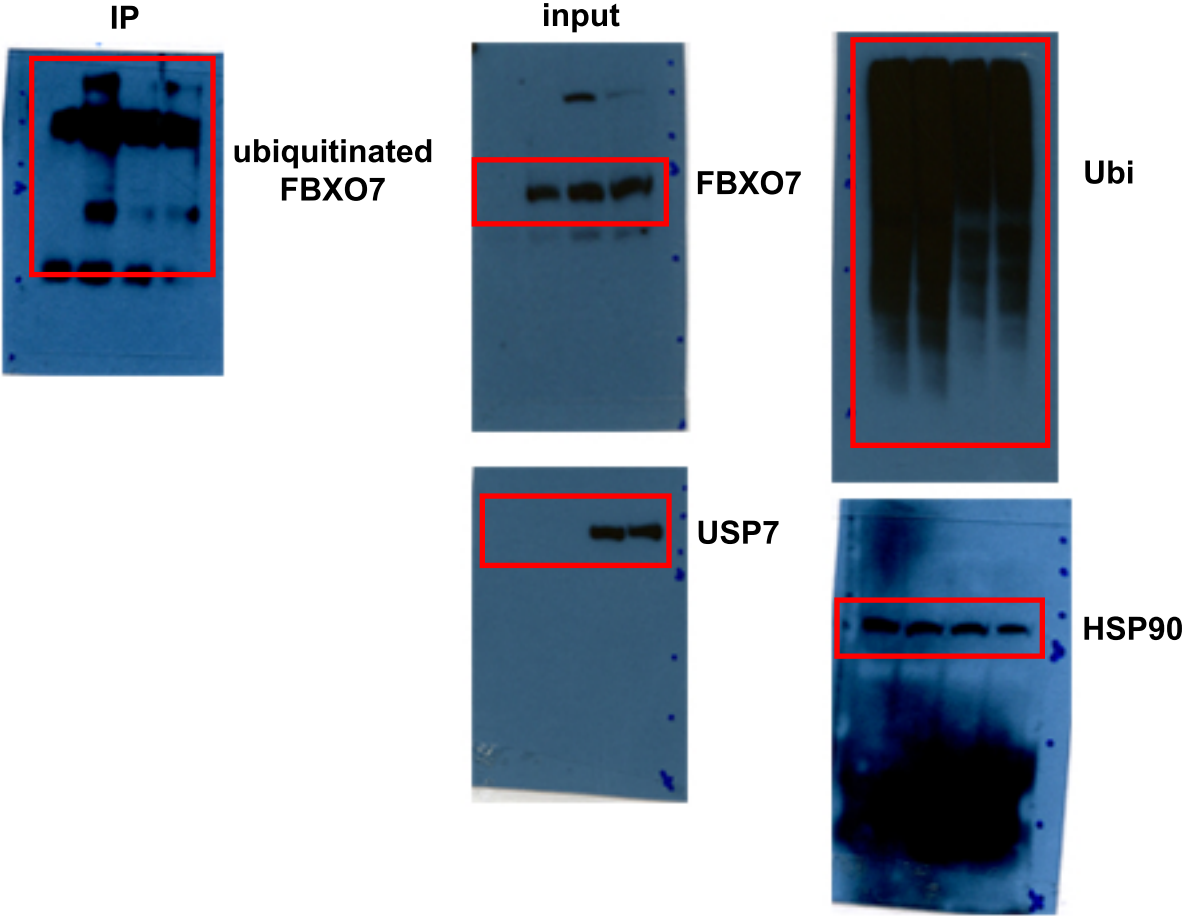

Supplement: S3 Raw image — (PDF) [file pone.0290371.s007.pdf]

Figure S4.

A

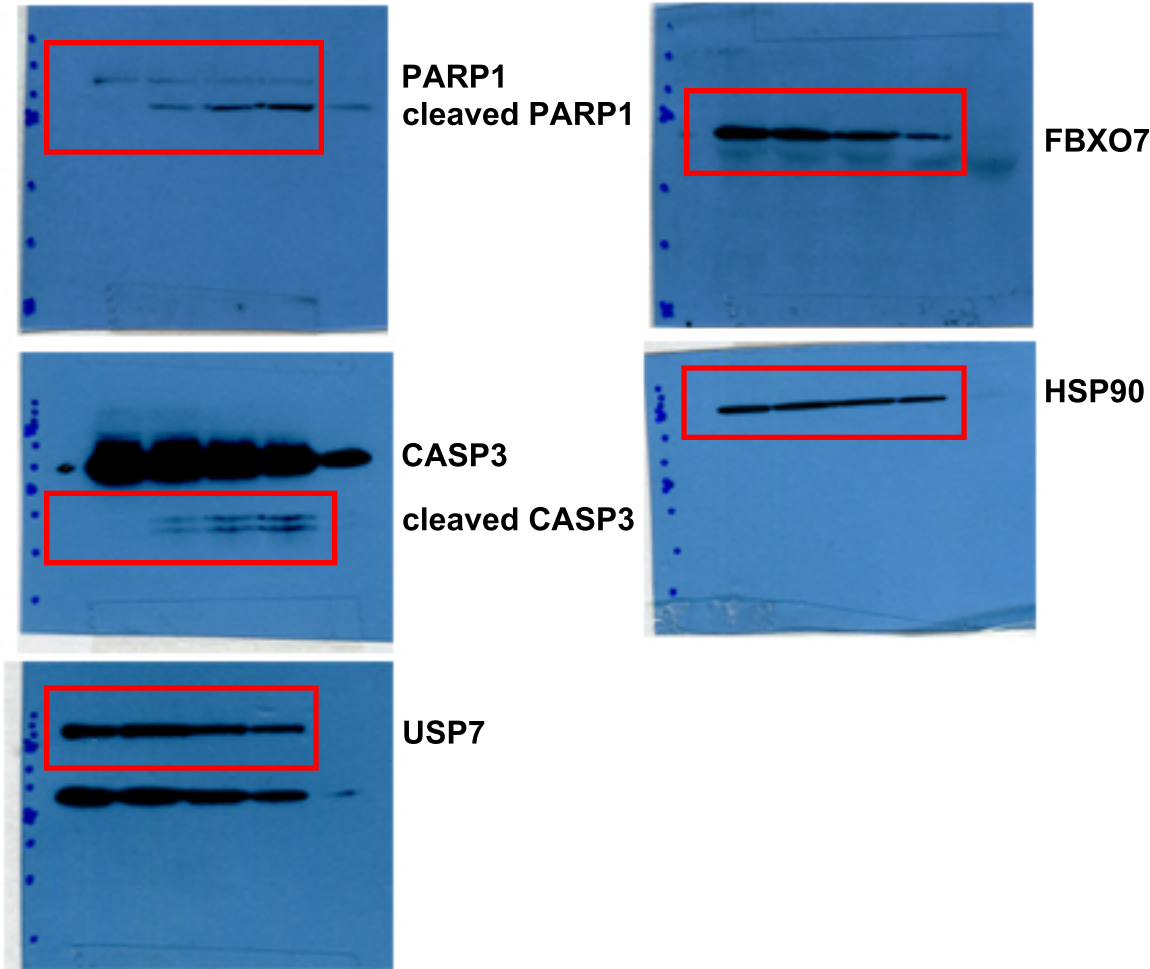

B

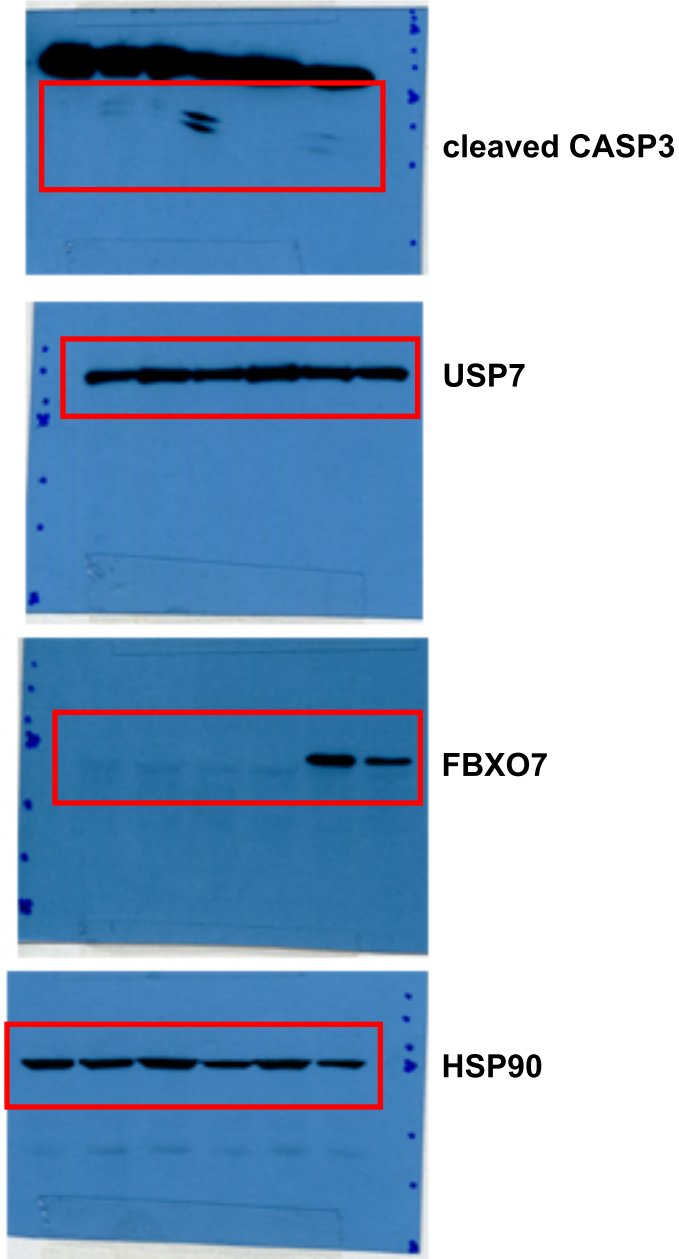

Supplement: S4 Raw image — (PDF) [file pone.0290371.s008.pdf]

**Figure 1.**

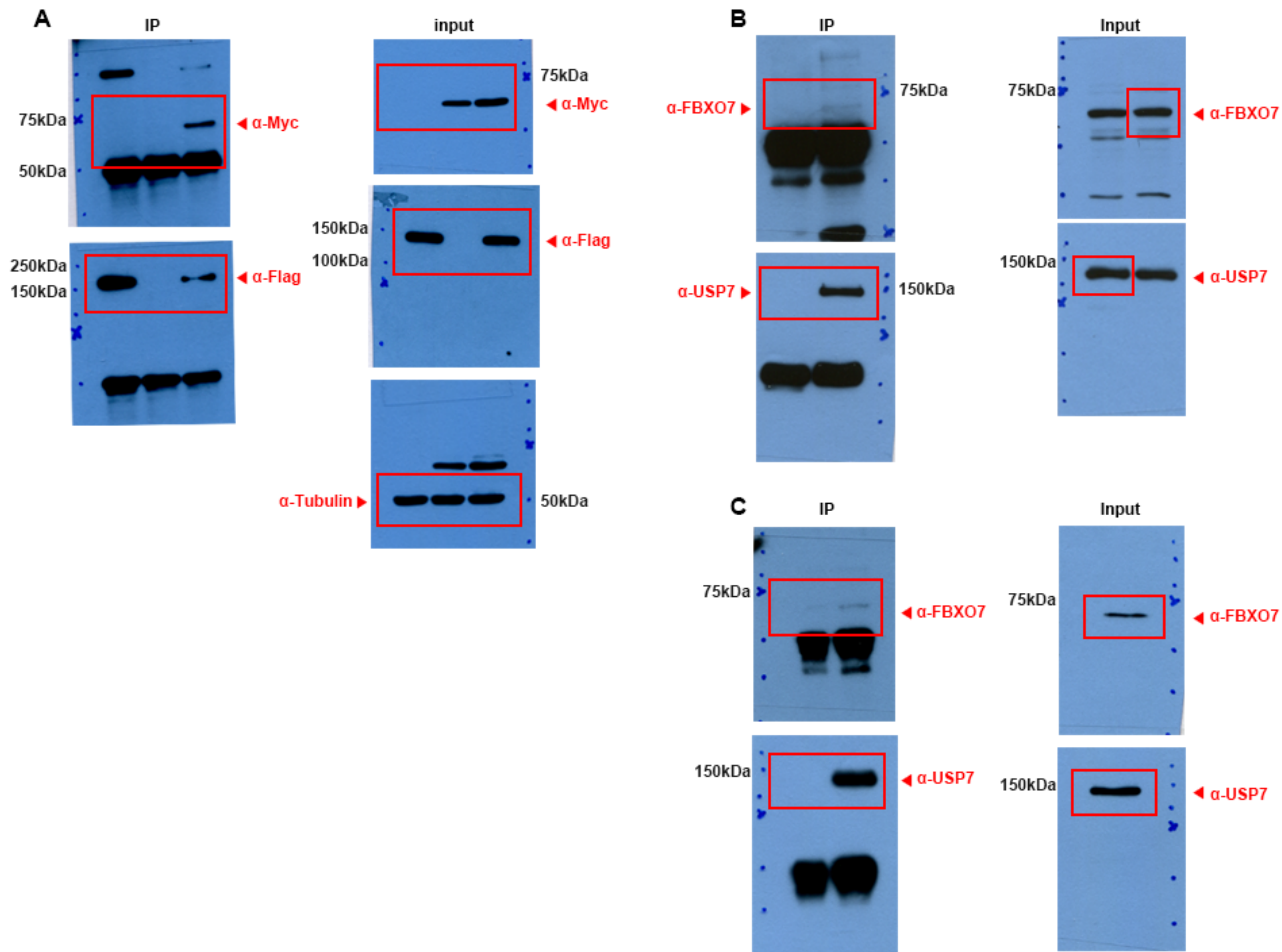

**Figure 2.**

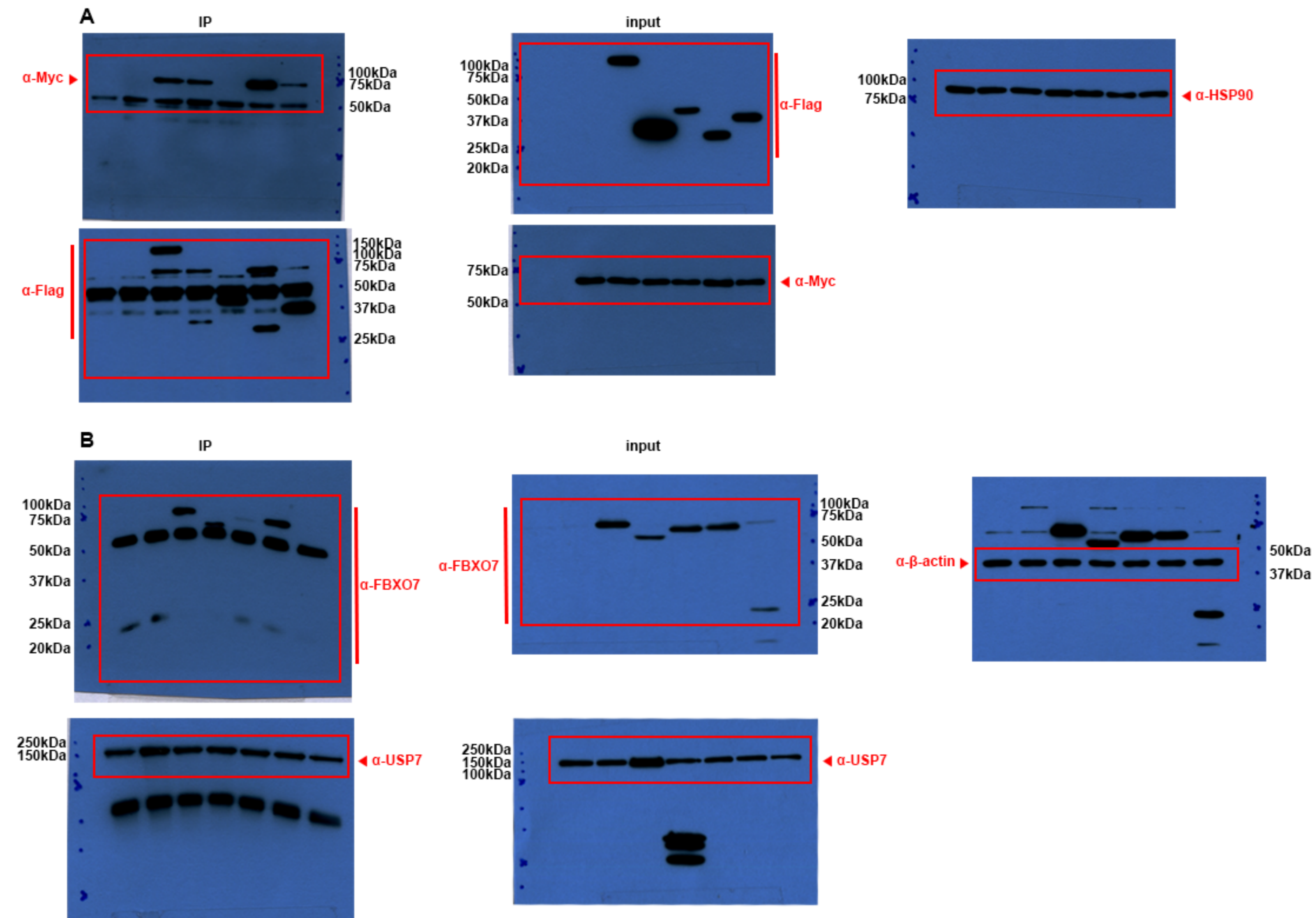

Figure 3.

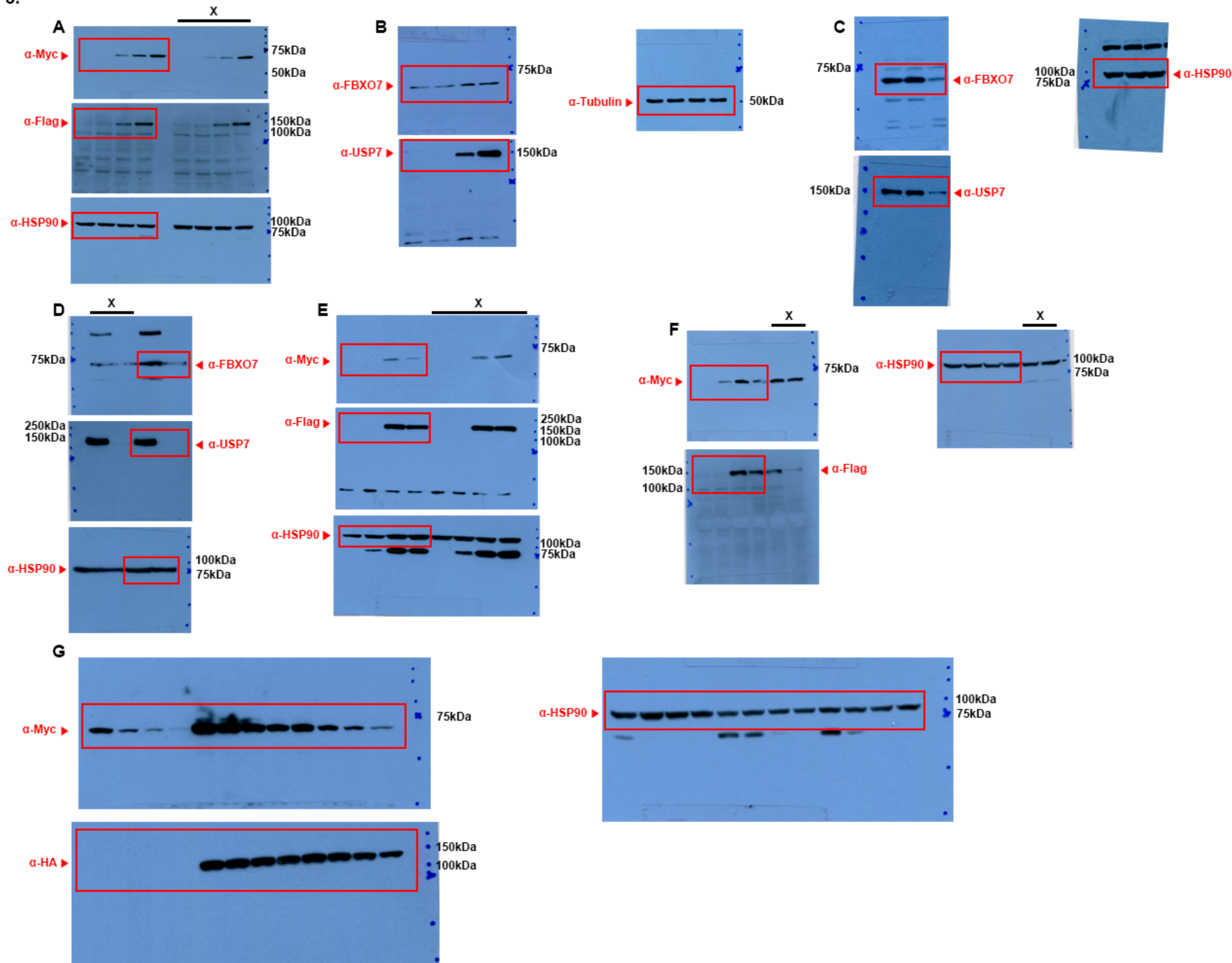

Figure 4.

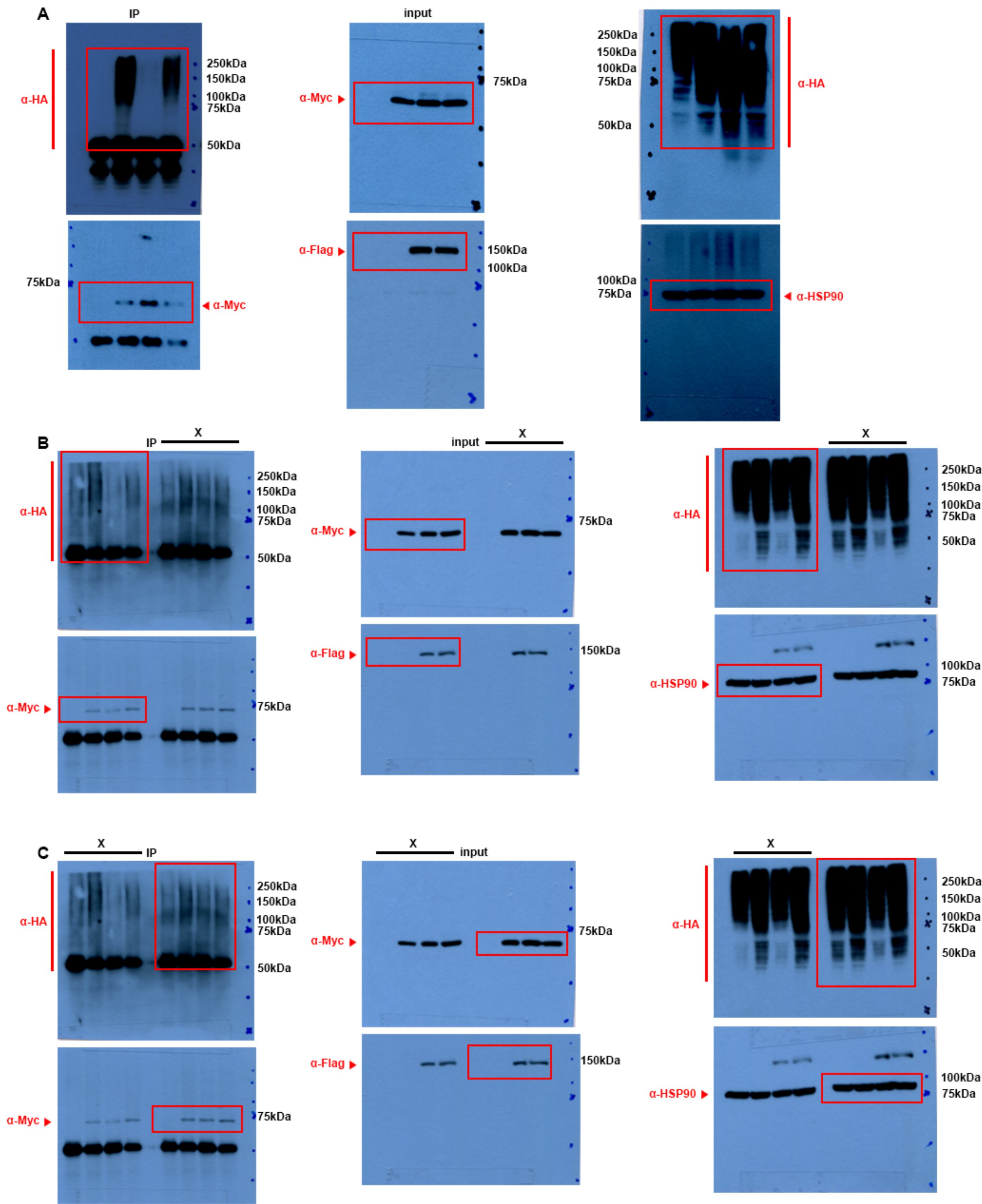

Figure 6.

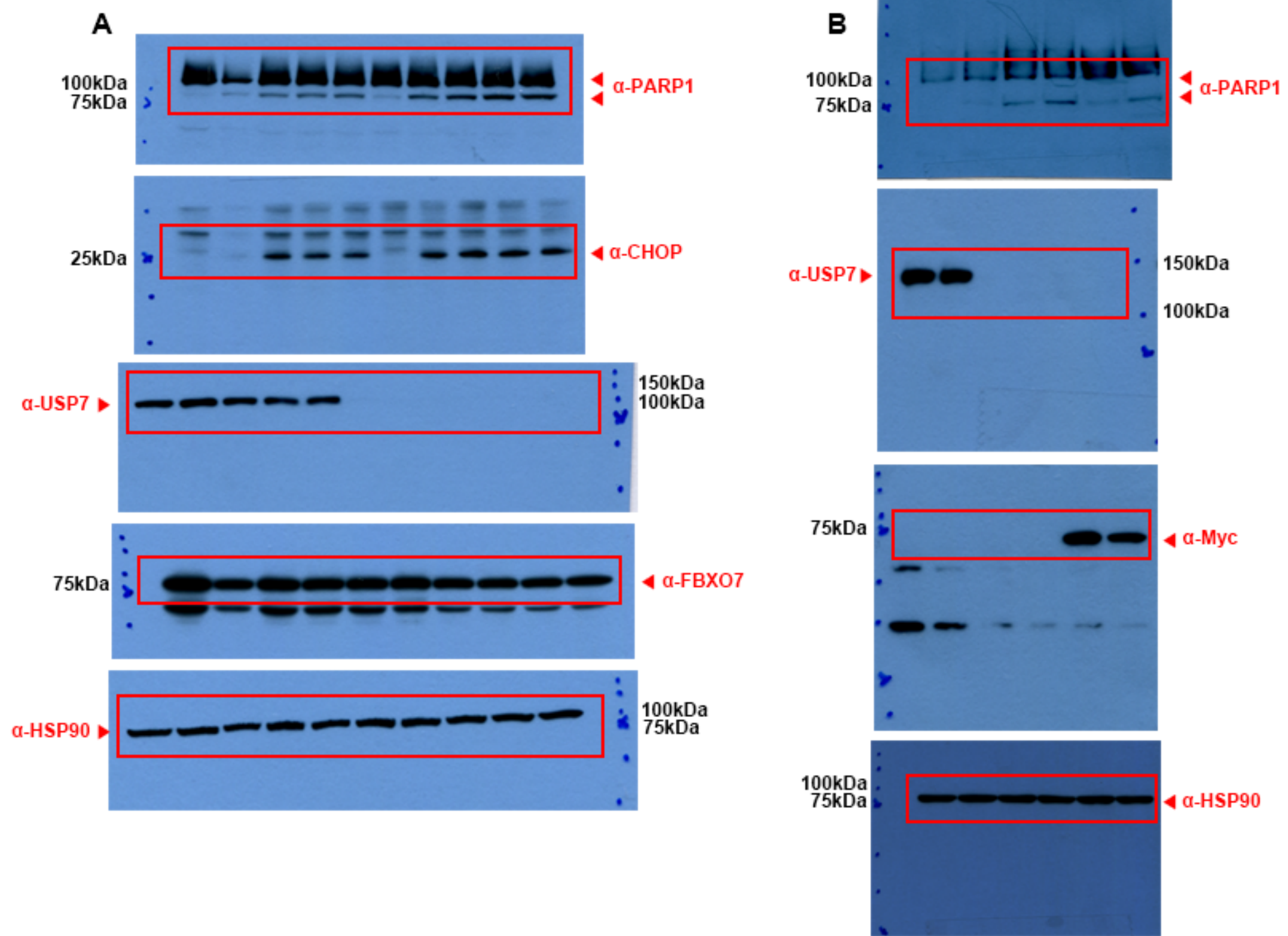

Figure 7.

**B**

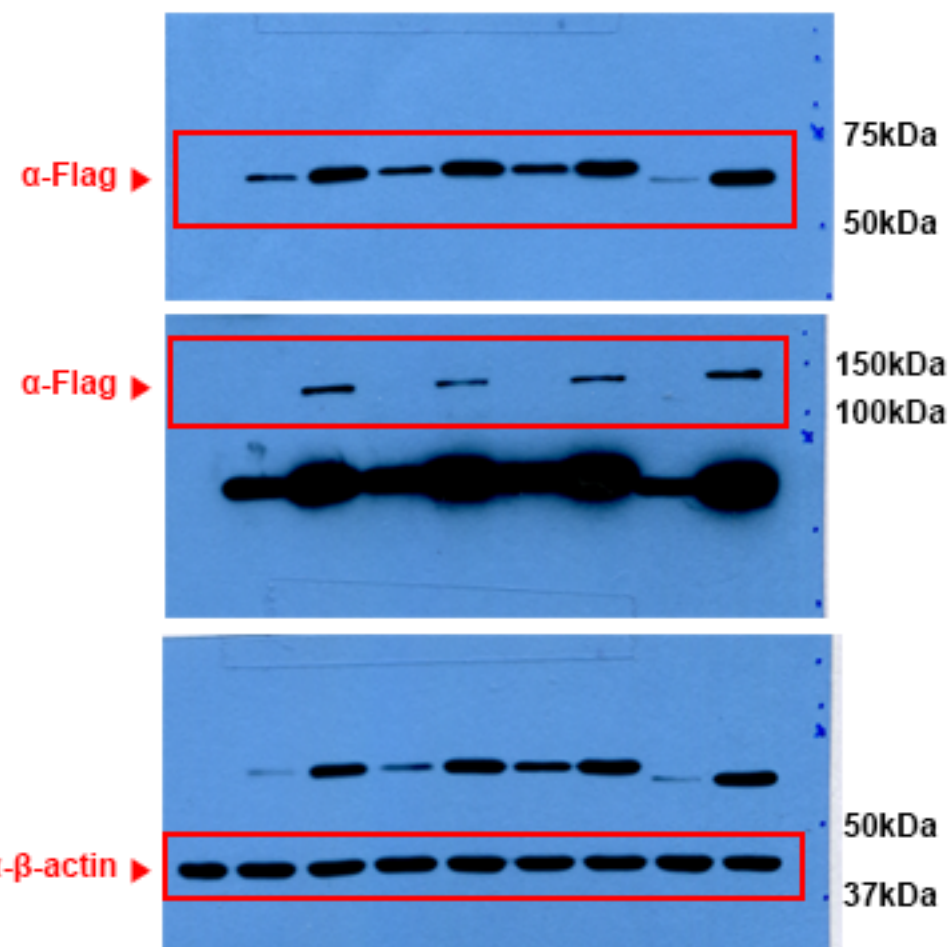

**C**

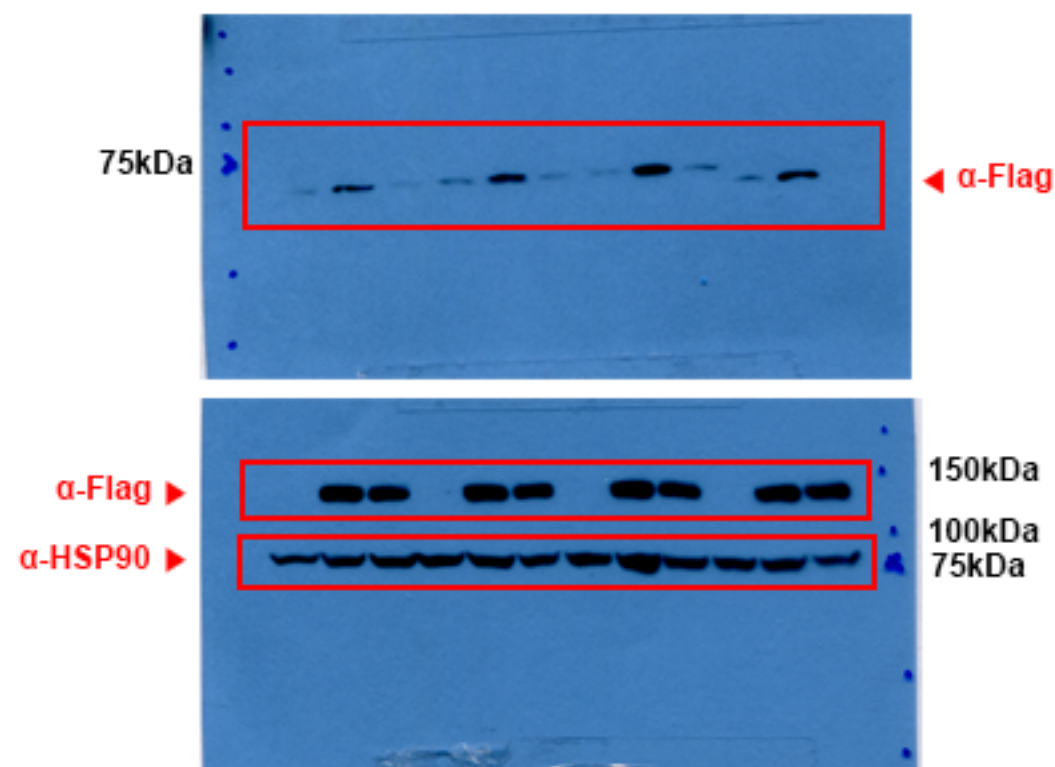

**D**

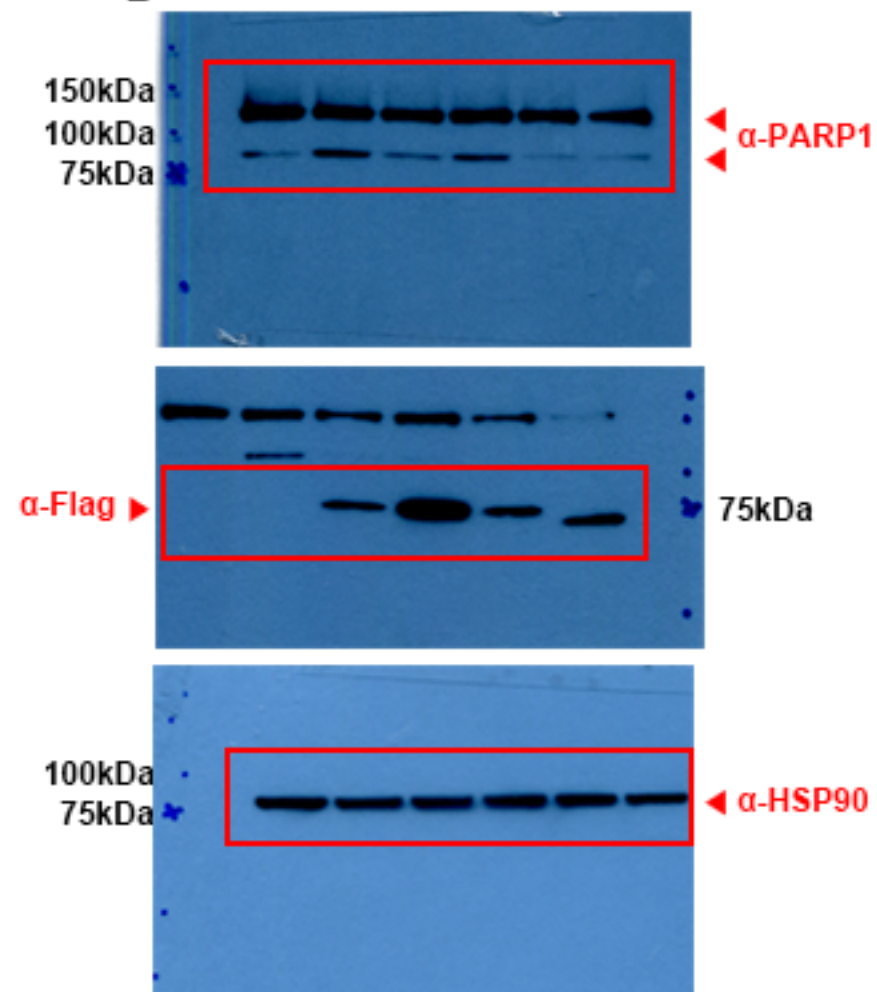

**Figure S1.**

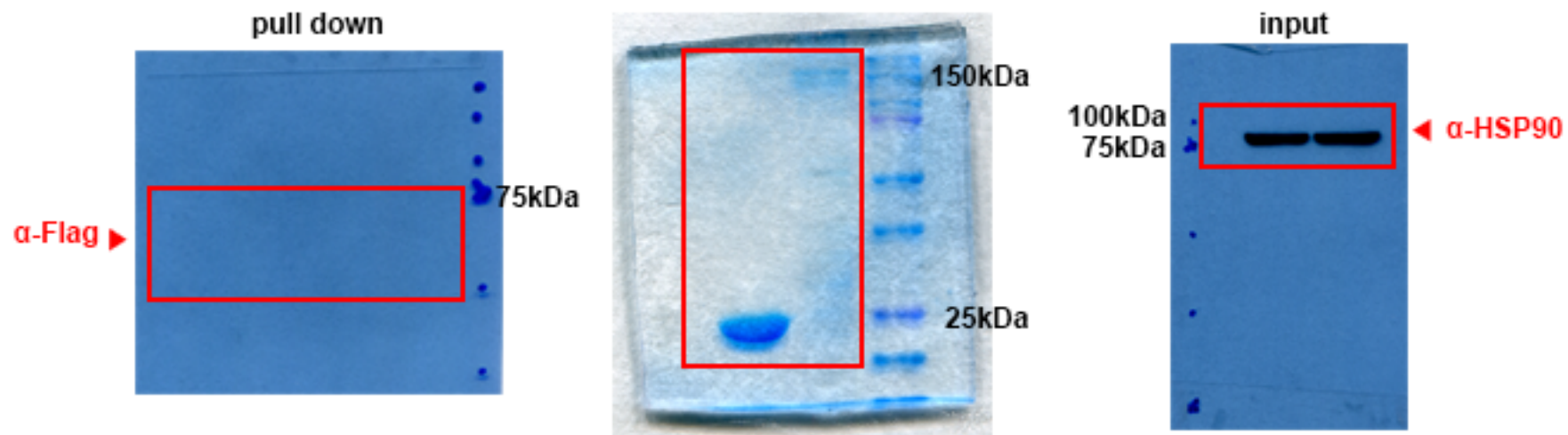

**Figure S2.**

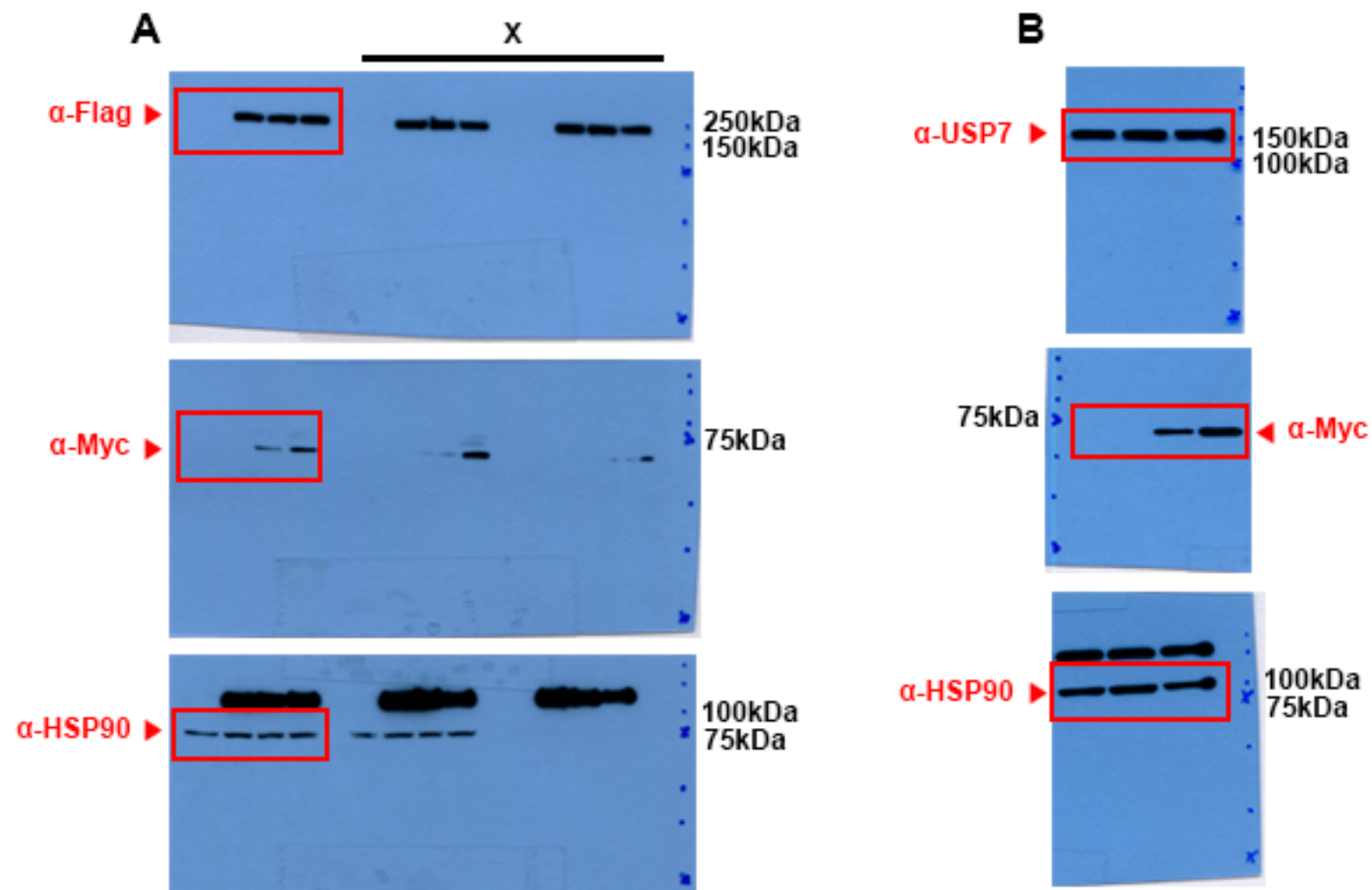

Figure S3.

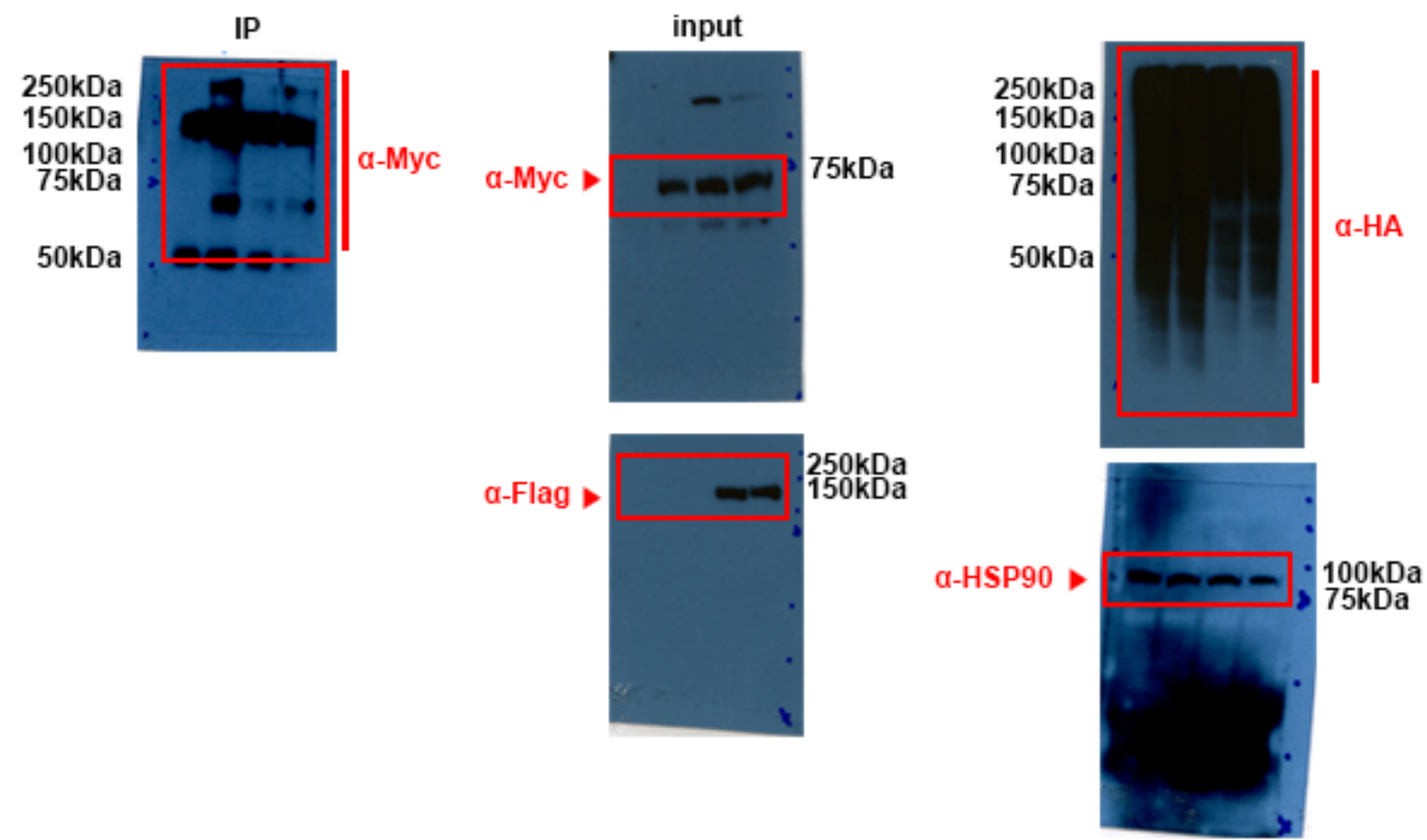

Figure S4.

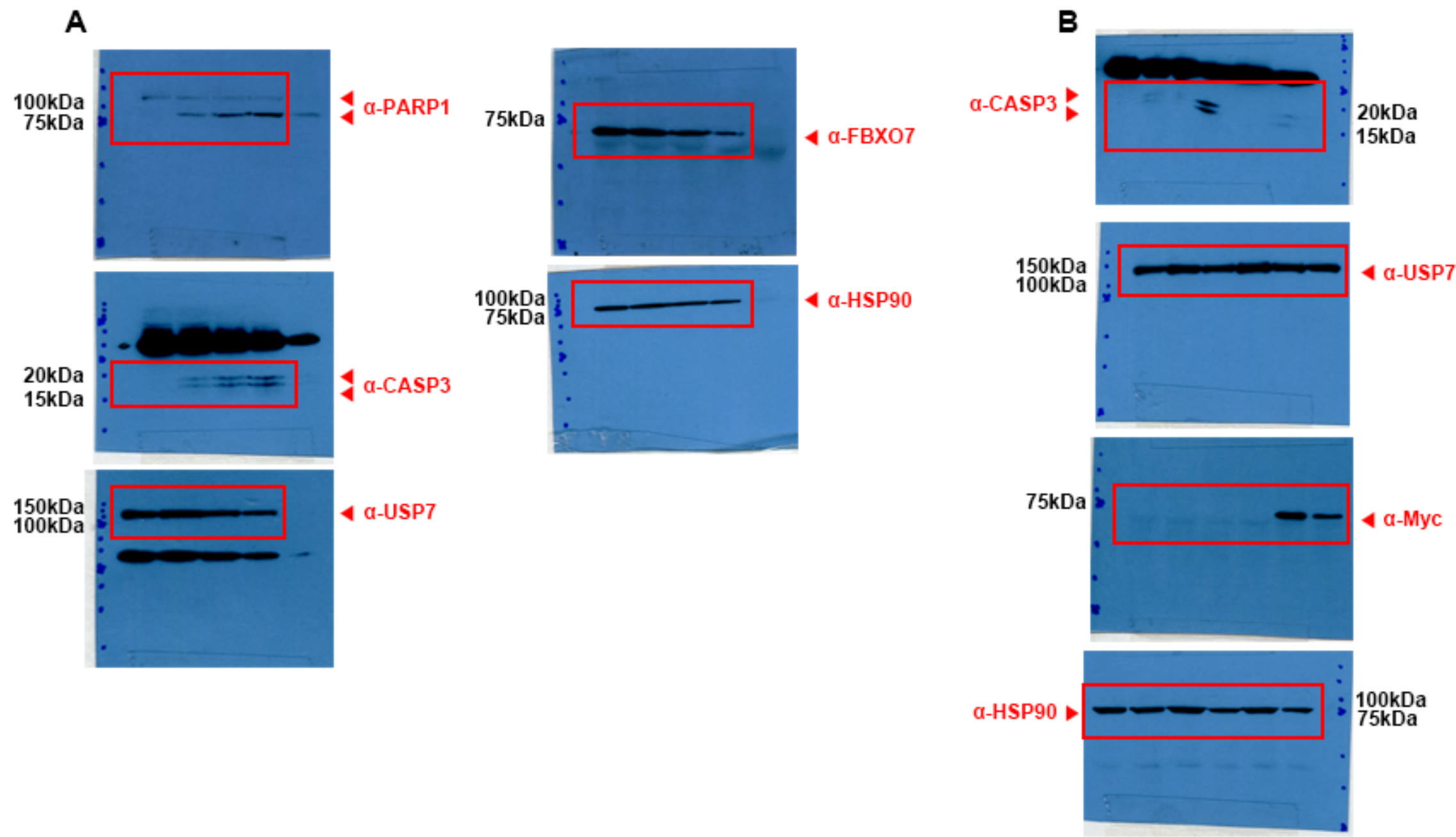

Supplement: S1 Data — (PDF) [file pone.0290371.s009.pdf]
