# Supplementary material for: USP7 attenuates endoplasmic reticulum stress-induced apoptotic cell death through deubiquitination and stabilization of FBXO7
Source: PLoS One. 2023 Oct 24;18(10):e0290371. doi: 10.1371/journal.pone.0290371 (PMC10597484; doi:10.1371/journal.pone.0290371)
Supplement: S3 Data — (PDF) [file pone.0290371.s011.pdf]

USP7 +/+ + DMSO

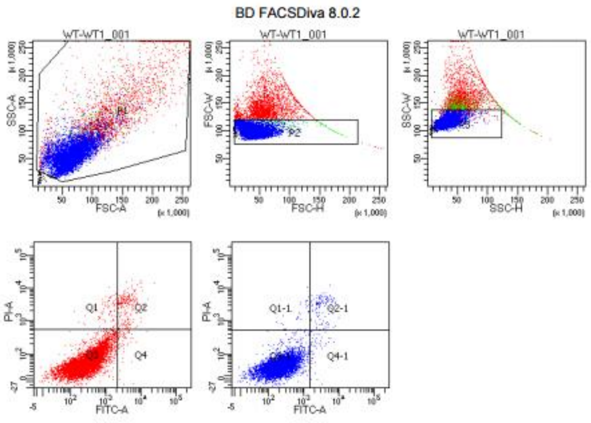

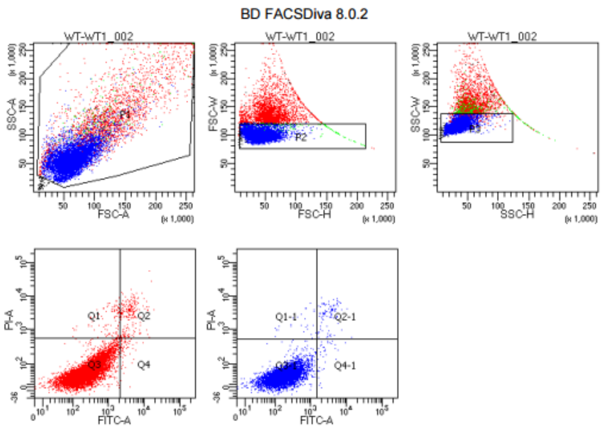

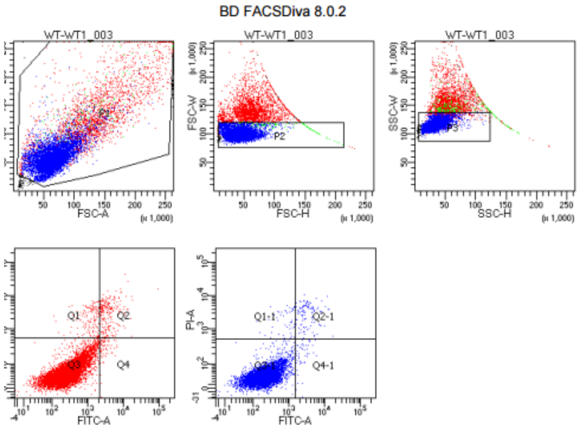

Tube: WT1_001

Population	#Events	%Parent	%Total
All Events	10,000	####	100.0
P1	9,871	98.7	98.7
P2	7,450	75.5	74.5
P3	7,201	96.7	72.0
Q1-1	34	0.5	0.3
Q2-1	118	1.6	1.2
Q3-1	7,000	97.2	70.0
Q4-1	49	0.7	0.5
Q1	85	0.9	0.9
Q2	239	2.4	2.4
Q3	9,472	96.0	94.7
Q4	75	0.8	0.8

Tube: WT1_002

Population	#Events	%Parent	%Total
All Events	10,000	####	100.0
P1	9,888	98.9	98.9
P2	7,306	73.9	73.1
P3	7,022	96.1	70.2
Q1-1	27	0.4	0.3
Q2-1	102	1.5	1.0
Q3-1	6,855	97.6	68.6
Q4-1	38	0.5	0.4
Q1	100	1.0	1.0
Q2	217	2.2	2.2
Q3	9,504	96.1	95.0
Q4	67	0.7	0.7

Tube: WT1_003

Population	#Events	%Parent	%Total
All Events	10,000	####	100.0
P1	9,852	98.5	98.5
P2	7,264	73.7	72.6
P3	7,022	96.7	70.2
Q1-1	49	0.7	0.5
Q2-1	107	1.5	1.1
Q3-1	6,819	97.1	68.2
Q4-1	47	0.7	0.5
Q1	145	1.5	1.5
Q2	235	2.4	2.4
Q3	9,395	95.4	94.0
Q4	77	0.8	0.8

	1	2	3
Q2-1	1.6	1.5	1.5
Q4-1	0.7	0.5	0.7
Q2-1 + Q4-1	**2.3**	**2**	**2.2**

USP7 +/+ + TM

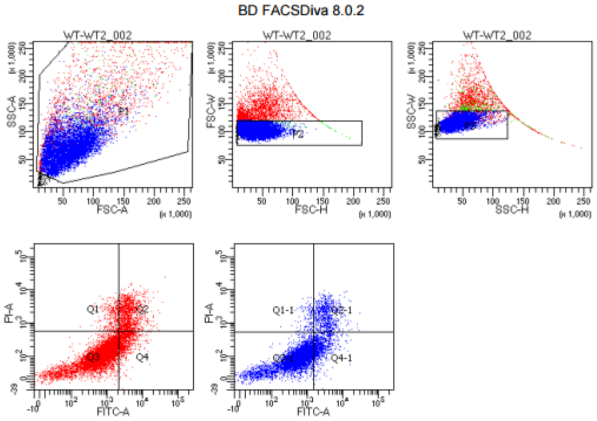

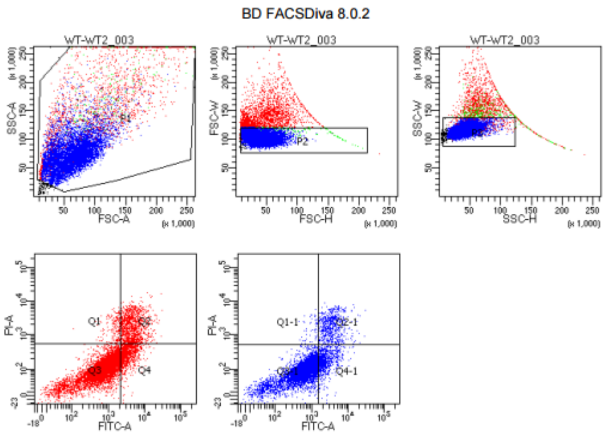

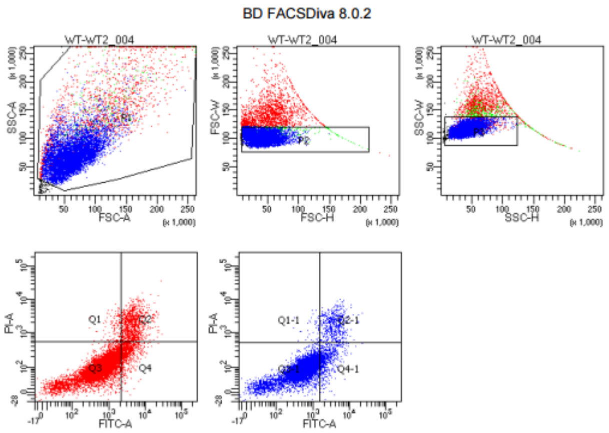

Tube: WT2_002

Population	#Events	%Parent	%Total
All Events	10,000	####	100.0
P1	9,820	98.2	98.2
P2	7,734	78.8	77.3
P3	7,539	97.5	75.4
Q1-1	113	1.5	1.1
Q2-1	706	9.4	7.1
Q3-1	5,793	76.8	57.9
Q4-1	927	12.3	9.3
Q1	297	3.0	3.0
Q2	1,244	12.7	12.4
Q3	7,384	75.2	73.8
Q4	895	9.1	9.0

Tube: WT2_003

Population	#Events	%Parent	%Total
All Events	10,000	####	100.0
P1	9,835	98.4	98.4
P2	7,673	78.0	76.7
P3	7,419	96.7	74.2
Q1-1	55	0.7	0.5
Q2-1	643	8.7	6.4
Q3-1	5,747	77.5	57.5
Q4-1	974	13.1	9.7
Q1	197	2.0	2.0
Q2	1,264	12.9	12.6
Q3	7,272	73.9	72.7
Q4	1,102	11.2	11.0

Tube: WT2_004

Population	#Events	%Parent	%Total
All Events	10,000	####	100.0
P1	9,876	98.8	98.8
P2	7,871	79.7	78.7
P3	7,587	96.4	75.9
Q1-1	43	0.6	0.4
Q2-1	499	6.6	5.0
Q3-1	6,300	83.0	63.0
Q4-1	745	9.8	7.4
Q1	131	1.3	1.3
Q2	1,033	10.5	10.3
Q3	7,884	79.8	78.8
Q4	828	8.4	8.3

	1	2	3
Q2-1	9.4	8.7	6.6
Q4-1	12.3	13.1	9.8
Q2-1 + Q4-1	**21.7**	**21.8**	**16.4**

USP7 -/- + DMSO

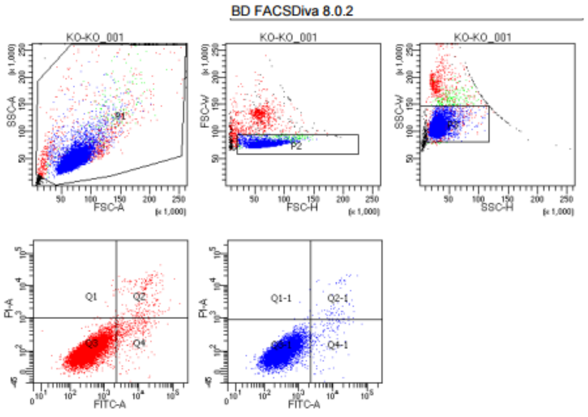

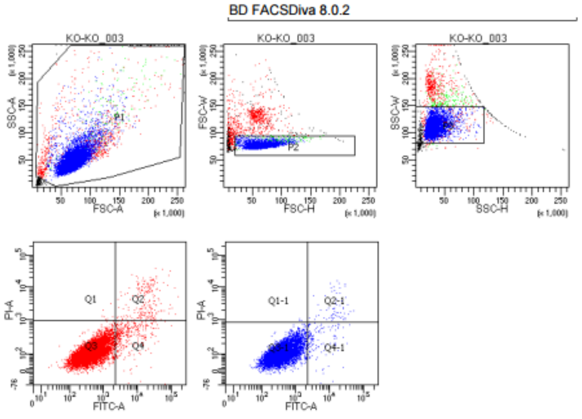

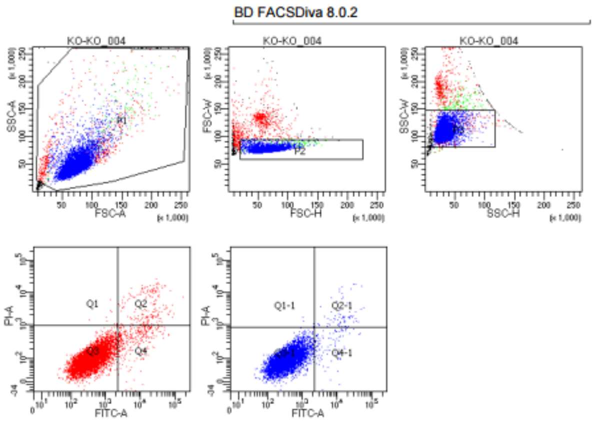

Tube: KO_001

Population	#Events	%Parent	%Total
All Events	10,000	####	100.0
P1	9,679	96.8	96.8
P2	8,814	91.1	88.1
P3	8,650	98.1	86.5
Q1-1	7	0.1	0.1
Q2-1	72	0.8	0.7
Q3-1	8,432	97.5	84.3
Q4-1	139	1.6	1.4
Q1	13	0.1	0.1
Q2	226	2.3	2.3
Q3	9,080	93.8	90.8
Q4	360	3.7	3.6

Tube: KO_003

Population	#Events	%Parent	%Total
All Events	10,000	####	100.0
P1	9,704	97.0	97.0
P2	8,887	91.6	88.9
P3	8,737	98.3	87.4
Q1-1	3	0.0	0.0
Q2-1	64	0.7	0.6
Q3-1	8,536	97.7	85.4
Q4-1	134	1.5	1.3
Q1	7	0.1	0.1
Q2	228	2.3	2.3
Q3	9,087	93.6	90.9
Q4	382	3.9	3.8

Tube: KO_004

Population	#Events	%Parent	%Total
All Events	10,000	####	100.0
P1	9,776	97.8	97.8
P2	9,018	92.2	90.2
P3	8,878	98.4	88.8
Q1-1	4	0.0	0.0
Q2-1	74	0.8	0.7
Q3-1	8,683	97.8	86.8
Q4-1	117	1.3	1.2
Q1	10	0.1	0.1
Q2	201	2.1	2.0
Q3	9,208	94.2	92.1
Q4	357	3.7	3.6

	1	2	3
Q2-1	0.8	0.7	0.8
Q4-1	1.6	1.5	1.3
Q2-1 + Q4-1	**2.4**	**2.2**	**2.1**

USP7 -/- + TM

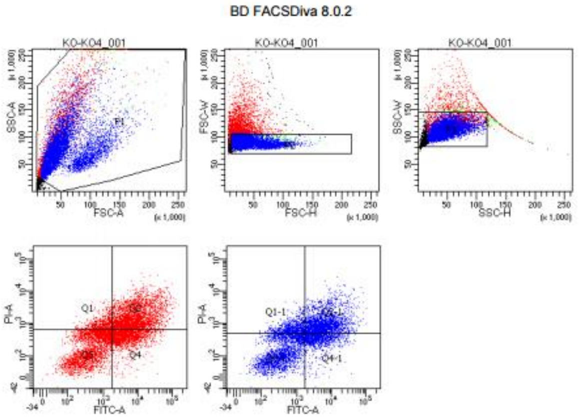

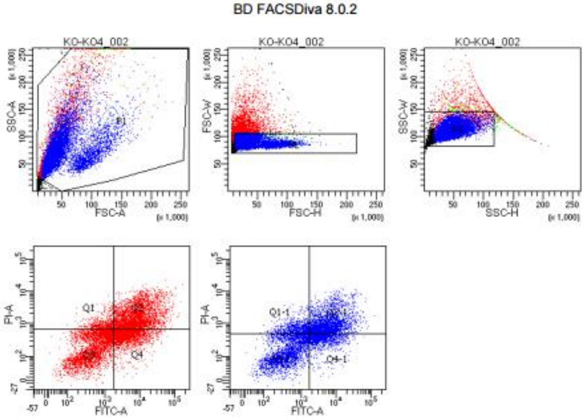

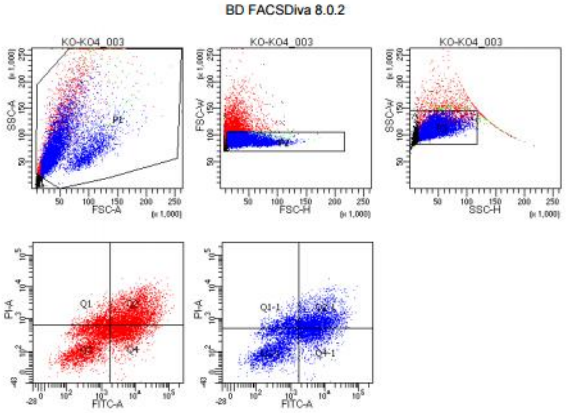

Tube: KO4_001

Population	#Events	%Parent	%Total
All Events	10,000	####	100.0
P1	8,978	89.8	89.8
P2	6,723	74.9	67.2
P3	6,626	98.6	66.3
Q1-1	759	11.5	7.6
Q2-1	2,311	34.9	23.1
Q3-1	2,305	34.8	23.0
Q4-1	1,251	18.9	12.5
Q1	623	6.9	6.2
Q2	3,260	36.3	32.6
Q3	3,017	33.6	30.2
Q4	2,078	23.1	20.8

Tube: KO4_002

Population	#Events	%Parent	%Total
All Events	10,000	####	100.0
P1	9,141	91.4	91.4
P2	7,050	77.1	70.5
P3	6,936	98.4	69.4
Q1-1	763	11.0	7.6
Q2-1	2,430	35.0	24.3
Q3-1	2,371	34.2	23.7
Q4-1	1,372	19.8	13.7
Q1	623	6.8	6.2
Q2	3,292	36.0	32.9
Q3	3,030	33.1	30.3
Q4	2,196	24.0	22.0

Tube: KO4_003

Population	#Events	%Parent	%Total
All Events	10,000	####	100.0
P1	8,672	86.7	86.7
P2	6,511	75.1	65.1
P3	6,419	98.6	64.2
Q1-1	651	10.1	6.5
Q2-1	2,234	34.8	22.3
Q3-1	2,137	33.3	21.4
Q4-1	1,397	21.8	14.0
Q1	595	6.9	5.9
Q2	2,986	34.4	29.9
Q3	2,822	32.5	28.2
Q4	2,269	26.2	22.7

	1	2	3
Q2-1	34.9	35	34.8
Q4-1	18.9	19.8	21.8
Q2-1 + Q4-1	**53.8**	**54.8**	**56.6**

USP7 -/- + FBXO7-WT + DMSO

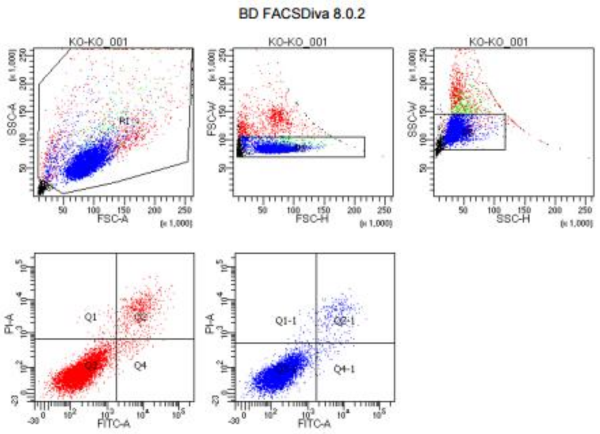

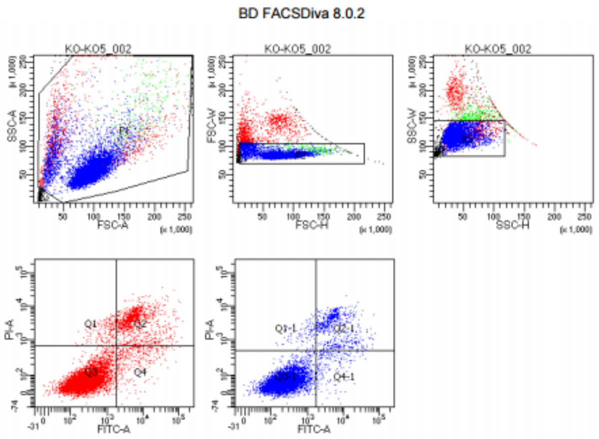

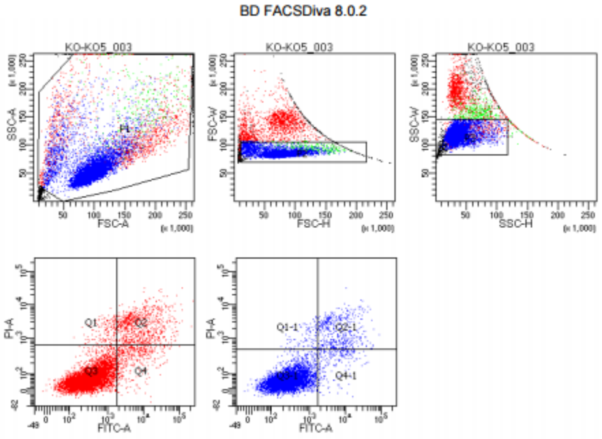

Tube: KO_001

Population	#Events	%Parent	%Total
All Events	10,000	####	100.0
P1	9,310	93.1	93.1
P2	8,419	90.4	84.2
P3	8,219	97.6	82.2
Q1-1	31	0.4	0.3
Q2-1	228	2.8	2.3
Q3-1	7,902	96.1	79.0
Q4-1	58	0.7	0.6
Q1	45	0.5	0.4
Q2	520	5.6	5.2
Q3	8,630	92.7	86.3
Q4	115	1.2	1.2

Tube: KO5_002

Population	#Events	%Parent	%Total
All Events	10,000	####	100.0
P1	9,609	96.1	96.1
P2	8,341	86.8	83.4
P3	8,040	96.4	80.4
Q1-1	108	1.3	1.1
Q2-1	686	8.5	6.9
Q3-1	7,042	87.6	70.4
Q4-1	204	2.5	2.0
Q1	212	2.2	2.1
Q2	1,282	13.3	12.8
Q3	7,750	80.7	77.5
Q4	365	3.8	3.6

Tube: KO5_003

Population	#Events	%Parent	%Total
All Events	9,859	####	100.0
P1	9,221	93.5	93.5
P2	7,652	83.0	77.6
P3	7,320	95.7	74.2
Q1-1	98	1.3	1.0
Q2-1	477	6.5	4.8
Q3-1	6,521	89.1	66.1
Q4-1	224	3.1	2.3
Q1	166	1.8	1.7
Q2	865	9.4	8.8
Q3	7,746	84.0	78.6
Q4	444	4.8	4.5

	1	2	3
Q2-1	2.8	8.5	6.5
Q4-1	0.7	0.5	3.1
Q2-1 + Q4-1	**3.5**	**11**	**9.6**

USP7 -/- + FBXO7-WT + TM

BD FACSDiva 8.0.2

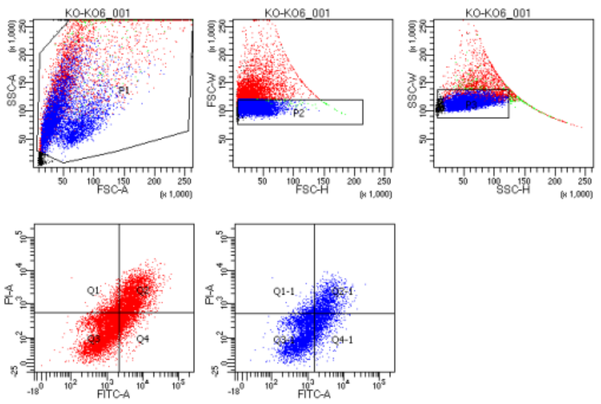

BD FACSDiva 8.0.2

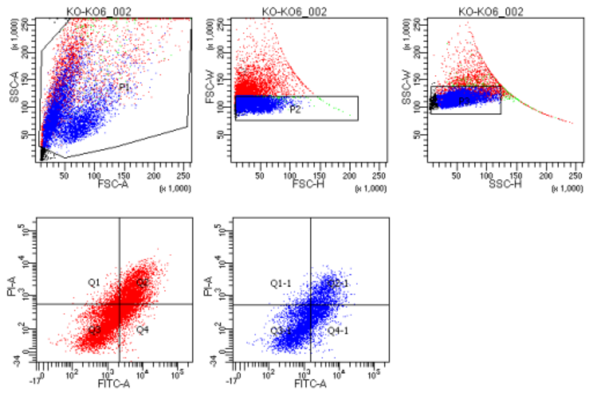

BD FACSDiva 8.0.2

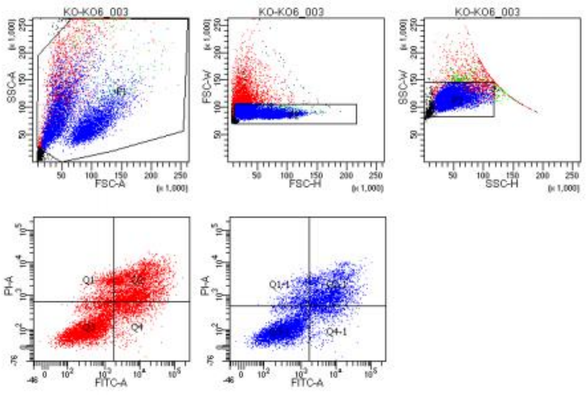

Tube: KO6_001

Population	#Events	%Parent	%Total
All Events	10,000	####	100.0
P1	9,538	95.4	95.4
P2	5,621	58.9	56.2
P3	5,459	97.1	54.6
Q1-1	224	4.1	2.2
Q2-1	1,227	22.5	12.3
Q3-1	2,894	53.0	28.9
Q4-1	1,114	20.4	11.1
Q1	638	6.7	6.4
Q2	3,259	34.2	32.6
Q3	4,389	46.0	43.9
Q4	1,252	13.1	12.5

Tube: KO6_002

Population	#Events	%Parent	%Total
All Events	10,000	####	100.0
P1	9,577	95.8	95.8
P2	5,936	62.0	59.4
P3	5,785	97.5	57.8
Q1-1	184	3.2	1.8
Q2-1	1,128	19.5	11.3
Q3-1	3,165	54.7	31.6
Q4-1	1,308	22.6	13.1
Q1	468	4.9	4.7
Q2	3,003	31.4	30.0
Q3	4,655	48.6	46.6
Q4	1,451	15.2	14.5

Tube: KO6_003

Population	#Events	%Parent	%Total
All Events	10,000	####	100.0
P1	9,143	91.4	91.4
P2	6,985	76.4	69.8
P3	6,794	97.3	67.9
Q1-1	538	7.9	5.4
Q2-1	2,002	29.5	20.0
Q3-1	3,572	52.6	35.7
Q4-1	682	10.0	6.8
Q1	649	7.1	6.5
Q2	3,237	35.4	32.4
Q3	4,072	44.5	40.7
Q4	1,185	13.0	11.8

	1	2	3
Q2-1	22.5	19.5	29.5
Q4-1	20.4	22.6	10
Q2-1 + Q4-1	**42.9**	**42.1**	**39.5**
