# Supplementary material for: Correction: USP7 attenuates endoplasmic reticulum stress-induced apoptotic cell death through deubiquitination and stabilization of FBXO7
Source: PLoS One. 2024 Jan 24;19(1):e0297970. doi: 10.1371/journal.pone.0297970 (PMC10807841; doi:10.1371/journal.pone.0297970)
Supplement: S1 File — (PDF) [file pone.0297970.s001.pdf]

PLOS ONE

RESEARCH ARTICLE

USP7 attenuates endoplasmic reticulum stress-induced apoptotic cell death through deubiquitination and stabilization of FBXO7

Su Hyoun Lee, Kwang Chul Chung *

Department of Systems Biology, College of Life Science and Biotechnology, Yonsei University, Seoul, Korea

* kchung@yonsei.ac.kr

OPEN ACCESS

Citation: Lee SH, Chung KC (2023) USP7 attenuates endoplasmic reticulum stress-induced apoptotic cell death through deubiquitination and stabilization of FBXO7. PLoS ONE 18(10): e0290371. https://doi.org/10.1371/journal.pone.0290371

Editor: Dipankar Ray, University of Michigan, UNITED STATES

Received: March 23, 2023

Accepted: August 7, 2023

Published: October 24, 2023

Copyright: © 2023 Lee, Chung. This is an open access article distributed under the terms of the Creative Commons Attribution License, which permits unrestricted use, distribution, and reproduction in any medium, provided the original author and source are credited.

Data Availability Statement: All relevant data are within the paper and its Supporting Information files.

Funding: This work was supported by a National Research Foundation of Korea (NRF) grant funded by the Ministry of Science and ICT, Korean government (NRF-2021R1A2C1005469 to K.C.C.). The funders had no role in study design, data collection and analysis, decision to publish, or preparation of the manuscript. There was no additional external funding received for this study.

Abstract

Parkinson's disease (PD) is a common neurodegenerative disease (NDD) characterized by the loss of dopaminergic neurons in the substantia nigra. Similar to other NDDs, the buildup of toxic protein aggregates in PD leads to progressive neuronal loss, culminating in neurodegeneration. Accumulating evidence indicates that alterations in subcellular organelles, particularly the endoplasmic reticulum (ER), are critically involved in pathological neurodegenerative events in NDDs, including PD. Mutations in the F-box only protein 7 (*FBXO7* or *PARK15*) gene have been found to cause early onset autosomal recessive familiar PD. FBXO7 functions as an adaptor protein in the Skp1-Cullin1-F-box protein (SCF) E3 ubiquitin ligase complex, which promotes substrate ubiquitination. Although FBXO7 is involved in the ubiquitination of various target proteins, little is known about the upstream regulatory mechanism of FBXO7 and/or its modulator(s). Ubiquitin specific protease 7 (USP7) is a deubiquitinating enzyme that regulates the balance between protein synthesis and degradation by removing ubiquitin from target substrates. The role of USP7 in various types of cancer is well-established; however, its role in NDDs has not been elucidated to date. In this study, we identified that USP7 acts as a novel regulator of FBXO7, positively regulating the stability of FBXO7 through Lys48-linked deubiquitination. Moreover, USP7 was found to mitigate ER stress-induced cytotoxicity and apoptosis by preventing the proteasomal degradation of FBXO7. Taken together, our study suggests that the functional relationship between FBXO7 and USP7 may play a crucial role in ER stress-induced apoptosis and the pathogenesis of PD.

Introduction

Parkinson's disease (PD) is the second most common neurodegenerative disease after Alzheimer's disease, which is caused by dopaminergic neuron loss in the substantia nigra of the midbrain [1]. There are several recognized risk factors for PD, including aging, abnormal and toxic protein aggregation, and mitochondrial dysfunction. Approximately 5–10% of PD cases are caused by mutations in one of several familiar genes such as α-synuclein, parkin, PTEN-induced kinase 1 (*PINK1*), *DJ-1*, *LRRK2*, *ATP13A2*, and F-box only protein 7 (*FBXO7*) [2, 3].

Competing interests: The authors have declared that no competing interests exist.

Abbreviations: CHOP, C/EBP homologous protein; DUB, deubiquitinating enzyme; co-IP, co-immunoprecipitation; DMEM, Dulbecco's modified Eagle's medium; ECL, enhanced chemiluminescence; ER, endoplasmic reticulum; ERAD, ER-associated protein degradation; FACS, fluorescence-activated cell sorter; FBS, fetal bovine serum; FBXO7, F-box only protein 7; HEK293, human embryonic kidney 293; HRP, horseradish peroxidase; LDH, lactate dehydrogenase; NDD, neurodegenerative disease; PARP1, poly(ADP-ribose) polymerase-1; PBS, phosphate-buffered saline; PD, Parkinson's disease; PEI, polyethylenimine; PI, propidium iodide; PINK1, PTEN-induced kinase 1; SCF, Skp1-Cullin-1-F-box; SD, standard deviation; TM, tunicamycin; Ubl, ubiquitin-like; UPR, unfolded protein response; UPS, ubiquitin proteasome system; USP, Ubiquitin-specific protease; USP7, ubiquitin-specific protease 7.

FBXO7 is a member of the F-box protein family and functions as an adaptor protein of the Skp1-Cullin1-F-box protein (SCF) ubiquitin E3 ligase complex [4]. While facilitating the ubiquitination of various substrates, FBXO7 also regulates several proteins in an SCF complex-independent manner [5–9]. For example, FBXO7 negatively regulates SIRT7 stability through lysine (Lys)48-linked polyubiquitination in an SCF-dependent manner [10]. Moreover, FBXO7 promotes the proteolysis of FOXO4 through SCF-independent pathways, including caspase-8 activation [11]. Although many substrates of the SCF complex and their pathophysiological roles have been well-identified, less is known about the upstream regulator(s) of FBXO7 and its underlying molecular mechanism remains to be fully elucidated.

The endoplasmic reticulum (ER) is an essential subcellular organelle with a variety of cellular functions, including the manufacturing and transport of lipids and proteins, proper protein folding and modification, and calcium homeostasis [12, 13]. When the environmental or physiological condition of cells change under various stimuli, including hypoxia, oxidative stress, and calcium depletion, aberrant proteins accumulate in the ER, resulting in a condition called ER stress [13]. As a protective response to ER stress, cells activate the unfolded protein response (UPR), accelerating the protein quality control of the ER [14]. However, when ER stress is excessive or persistent, ER function is disturbed, resulting in the accumulation of unfolded and misfolded proteins in the ER. Continuous activation of the UPR eventually becomes cytotoxic and initiates apoptosis [15–18]. Recent studies have suggested a close link between ER stress and PD-associated pathologies [19].

Deubiquitinating enzymes (DUBs) remove ubiquitin from target proteins, which can either rescue proteins from degradation or modulate their functional activity or signaling [20]. Approximately 100 DUBs are encoded in the human genome, which are subdivided into seven families, according to their catalytic domains: Ubiquitin-specific proteases (USPs), ubiquitin C-terminal hydrolases, ovarian tumor proteases, Machado-Joseph disease proteases, Jab1/Mov34/Mpr1 metalloproteases, MIU-containing novel DUB, and zinc finger-containing ubiquitin peptidase 1 [20]. USP7, also known as herpes virus-associated ubiquitin-specific protease (HAUSP), is a deubiquitinating enzyme that modifies ubiquitin on its substrates and thus affects various cellular processes such as the stress response, apoptosis, and the cell cycle [21, 22]. For example, USP7 removes the Lys63-linked polyubiquitin chains of SIRT7 as well as a single ubiquitin moiety from FOXO4 [23, 24]. Unlike polyubiquitinated targets via Lys48 linkage that are ultimately destined for proteasomal degradation, USP7 only negatively regulates the activities of SIRT7 and FOXO4 with no effect on their stability [23, 24].

Based on recent findings that USP7 and FBXO7 are involved in the regulation of SIRT7 and FOXO4, we hypothesized that there might be a biochemical and/or functional relationship between the two proteins. In this study, we found that USP7 interacts with FBXO7 in mammalian cells. In addition, USP7 prevented the proteasomal degradation of FBXO7 through Lys48-linked deubiquitination. Furthermore, *USP7* knockout or treatment of USP7-specific chemical inhibitors exacerbated tunicamycin (TM)-induced cytotoxicity and apoptosis, which was considerably mitigated by FBXO7 overexpression. Collectively, the present study implies that USP7 attenuates ER stress-induced apoptosis through the deubiquitination and stabilization of FBXO7, which may also play a role in PD pathogenesis.

Materials and methods

Materials

Dulbecco's modified Eagle's medium (DMEM), fetal bovine serum (FBS), Lipofectamine® 2000, polyethylenimine (PEI) reagent, and anti-mouse and anti-rabbit horseradish peroxidase (HRP)-conjugated secondary antibodies were purchased from PerkinElmer and Analytical

Sciences (Waltham, MA, USA). *Trans*IT-X2® Dynamic Delivery System for plasmid DNA and siRNA transfection was purchased from Mirus Bio (Madison, WI, USA). Protein-A Sepharose beads were purchased from GE Healthcare Biosciences (Piscataway, NJ, USA). TM and cycloheximide were purchased from Sigma-Aldrich (St. Louis, MO, USA). P5091 was purchased from Selleck Chemicals (Houston, TX, USA). Enhanced chemiluminescence (ECL) reagent was purchased from Abclon (Seoul, Korea) and Advansta (Menlo Park, CA, USA). All other chemicals used in the study were analytical-grade commercial products purchased from Sigma-Aldrich. The following primary antibodies (and their catalog numbers) for western blotting were purchased from the indicated vendors: Mouse monoclonal anti-HA (sc-7392, Santa Cruz Biotechnology), anti-Flag (F3165, Sigma-Aldrich), anti-Myc (sc-40, Santa Cruz Biotechnology), anti-FBXO7 (sc-271763, Santa Cruz Biotechnology), anti-C/EBP homologous protein (CHOP) (L63F7, Cell Signaling Technology), anti-HSP90 (sc-13119, Santa Cruz Biotechnology), rabbit monoclonal anti-USP7 (GTX125894, GeneTex), anti-poly(ADP-ribose) polymerase-1 (PARP1) (9542, Cell Signaling Technology), and anti-caspase 3 (9662, Cell Signaling Technology). HRP-conjugated anti-mouse (AP124P) and anti-rabbit (AP132P) secondary antibodies were purchased from EMD Millipore (Billerica, MA, USA).

DNA constructs and RNA interference

Mammalian expression vectors for Flag-tagged human wild-type FBXO7 isoform-1 and its F-box deletion mutant were provided by H.J. Kuiken (The Netherlands Cancer Institute, Amsterdam, Netherlands). The mammalian expression vectors for Flag-tagged human wild-type USP7 and its deletion mutants were provided by J.H. Kim (Inha University, Incheon, Korea). The bacterial plasmids encoding glutathione S-transferase (GST)-tagged USP7 (pGEX-4T-2-*USP7)* or GST as a control (pGEX-4T-2) were provided by K.H. Baek (CHA University, Gyeonggi-do, Korea). All plasmid sequences were verified by DNA sequencing (Bionics, Seoul, Korea). Small interfering RNAs (siRNAs) for *FBXO7* and *USP7*, and control scrambled siRNA (cat. #51-01-14-04) were designed and synthesized by Integrated DNA Technologies (Hanam-si, Gyeonggi-do, Korea). *USP7*-specific siRNA duplex sequences were `5′-AUCAGCAGCUUAAGAUGAAAAUCAC(dTdT)-3′` (sense) and `5′-GUGAUUUUCAUCUUAAGCUGCUGAUAG(dTdT)-3′` (antisense). *FBXO7*-specific siRNA duplex sequences were `5′-UUGGUUCUCCUCUAGAUUGAAGU(dTdT)-3′` (sense) and `5′-ACUUCAAUCUAGAGGAGAACCAA(dTdT)-3′` (antisense).

Cell culture and DNA transfection

Human embryonic kidney 293 (HEK293) cells were maintained in DMEM supplemented with 10% FBS and 100 U/mL penicillin-streptomycin. *USP7*-knockout HeLa cells were provided by S. Ramakrishna. (Hanyang University, Seoul, Korea) and maintained in Iscove's modified Dulbecco's medium supplemented with 10% FBS and 100 U/mL penicillin-streptomycin. Human neuroblastoma SH-SY5Y cells were maintained in DMEM/F12 supplemented with 10% FBS and 100 U/mL penicillin-streptomycin. All cells were grown at 37˚C with 5% $CO_{2,}$ unless otherwise indicated. All DNA transfections were carried out using PEI, Lipofectamine 2000, or Mirus reagent, according to the manufacturer's instructions.

Co-immunoprecipitation (Co-IP) and immunoblot analysis

Cell lysates were washed with ice-cold phosphate-buffered saline (PBS), scraped, and mixed with 1% Nonidet P-40 lysis buffer (50 mM Tris, pH 7.5, 1% Nonidet P-40, 150 mM NaCl, 10% glycerol, 1 mM sodium orthovanadate, 1 μg/mL aprotinin, 1 mM EGTA, 1 mM sodium fluoride, and 0.2 mM phenylmethylsulfonyl fluoride). The cells were sonicated and the

supernatants were collected by centrifugation at 13,000 × *g* for 15 min at 4˚C. For immunoprecipitation, 500~1000 μg of cell lysates were incubated with 1 μg of appropriate antibodies overnight at 4˚C with gentle rotation. Protein A Sepharose beads were then added and incubated for 2 h at 4˚C with gentle rotation. The beads were washed with 1% Nonidet P-40 lysis buffer, and the immunocomplexes were dissociated by boiling in 2× sample buffer. The samples were then resolved by sodium dodecyl sulfate-polyacrylamide gel electrophoresis, and transferred to a nitrocellulose membrane (Whatman, GE Healthcare Life Sciences). The membranes were blocked in Tris-buffered saline with Tween (TBST) buffer (20 mM Tris, pH 7.5, 137 mM NaCl, and 0.1% Tween 20) and 5% nonfat dry milk for 1 h at room temperature. Membranes were then incubated with the appropriate primary antibody at 4˚C overnight. The membranes were washed in TBST and incubated with HRP-conjugated secondary antibody for 2 h at room temperature. The membranes were washed again with TBST, and the protein bands were visualized using ECL reagents, according to the manufacturer's instructions.

Immunocytochemistry analysis

SH-SY5Y cells were seeded onto poly D-lysine-coated cover glasses. Adherent cells were washed twice with PBS and immediately fixed in 3.7% formaldehyde for 10 min at room temperature. After fixation, cells were permeabilized with 0.1% Triton X-100 for 10 min and blocked with 1% bovine serum albumin in TBST for 1 h at room temperature. Cells were then immunostained using rabbit polyclonal anti-USP7 or/and mouse monoclonal anti-FBXO7 antibodies, washed, and incubated with Alexa Fluor 488- or Alexa Fluor 594-conjugated anti-IgG antibodies. Images were captured using an LSM 880 confocal microscope (Carl Zeiss, Oberkochen, Germany) and processed using the Zeiss LSM Image Browser (Carl Zeiss).

In vitro GST pull-down assay

Bacterial plasmids encoding GST-USP7 or GST were transformed into *E.coli* BL21 cells. Their protein expressions were induced by adding 1 mM isopropyl 1-thio-β-D-galactopyranoside at 16˚C. The cells were harvested and lysed by sonication. The GST and GST-USP7 fusion protein were immobilized on glutathione-Sepharose 4B beads (GE Healthcare Biosciences), according to the supplier's instructions. HEK293 cells were transiently transfected for 24 h with Flag-FBXO7, harvested, and lysed in lysis buffer. HEK293 cell lysates containing Flag-FBXO7 protein were incubated with immobilized GST-tagged USP7 proteins overnight at 4˚C, and bound proteins were analyzed by western blotting.

Flow cytometry analysis

Cell apoptosis was detected using Annexin V-fluorescein isothiocyanate (FITC)/propidium iodide (PI) apoptosis staining/detection Kit (ab14085, Abcam), and fluorescence-activated cell sorting (FACS) was used for quantification. After ~1 x 10^5 cells were seeded in 6-well plates and treated with 1 μM TM or vehicle (dimethyl sulfoxide) for 24 h, cells were collected and resuspended in 500 μL 1X binding buffer, followed by staining with 5 μL Annexin V-FITC and 5 μL PI for 15 min at room temperature in the dark. The stained samples were analyzed using the BD FACS LSR II SORP system (Becton Dickinson). Theoretically, cells could be divided into the following four groups according to fluorescence staining through flow cytometry: non-apoptotic cells (Annexin V-FITC-positive/PI-negative), late apoptotic/necrotic cells (Annexin V-FITC-positive/PI-positive), and dead cells (Annexin V-FITC-negative/PI-positive).

Lactate dehydrogenase (LDH) cytotoxicity assays

Cytotoxicity was evaluated using an LDH Cytotoxicity Detection Kit (Takara, Kyoto, Japan). *USP7*-knockout HeLa and SH-SY5Y cells were transfected with DNA for 24 h, followed by treatment with 0.5 μM or 1 μM TM for an additional 24 h. Cell-free culture media were then collected and used in the LDH assay, according to the manufacturer's instructions. The maximum LDH release (referred to as "high control") was determined by solubilizing the cells in 1% Triton X-100. Spontaneous LDH release (referred to as "low control") was determined by incubating the cells in the medium alone. Absorbance was measured at 490 nm using a microplate reader. Cytotoxicity was calculated as a percentage of the control using the following formula: cytotoxicity = [(experimental value–low control)/(high control–low control)] × 100%.

Statistical analysis

Unpaired Student's *t*-tests were used for all statistical analyses to compare data from different groups. The analysis was performed using the GraphPad Prism software (version 5; San Diego, CA, USA). All values are reported as mean ± standard deviation of at least three independent experiments. The intensities of the western blot bands were measured using GelQuant.NET software (version 1.8.2).

Results

USP7 interacts with FBXO7 in mammalian cells

Numerous substrates of FBXO7 have been reported to date. We recently found that FBXO7 promotes the degradation of FOXO4 and SIRT7 [10, 11]. Previous studies further showed that the functional activities of both FOXO4 and SIRT7 are negatively regulated by USP7, without an influence on their stability [23, 24]. Based on these studies, we hypothesized that USP7 may be physically and/or functionally related to FBXO7. To test this hypothesis, we first examined the binding of FBXO7 to USP7 in mammalian cells. HEK293 cells were transfected with a plasmid encoding Myc-tagged FBXO7 alone or together with Flag/HA-USP7, and co-IP of cell lysates was performed using an anti-Flag antibody. Immunoblotting of anti-Flag immunocomplexes with an anti-Myc antibody revealed that ectopically expressed FBXO7 interacted with USP7 in HEK293 cells (Fig 1A). In addition, the interaction between endogenous FBXO7 and USP7 was confirmed in HEK293 and human neuroblastoma SH-SY5Y cells (Fig 1B and 1C). Furthermore, we investigated the direct binding of USP7 to FBXO7 through the utilization of a recombinant GST-USP7 in the GST pull-down assay. Interestingly, the results revealed that GST-USP7 does not interact with Flag-FBXO7 (S1 Fig). This result indicated that USP7 indirectly interacts with FBXO7 through the protein complex(s) or additional unknown target(s). Further study is necessary to clarify the exact mechanism of the interaction between USP7 and FBXO7. Immunohistochemical analysis of SH-SY5Y cells further revealed that endogenous FBXO7 and USP7 co-localized within cells (Fig 1D). Taken together, these results suggested that USP7 interacts with FBXO7 in mammalian cells.

Both the N- and C-terminal regions of USP7 bind to the central region of FBXO7

To determine which domain(s) of USP7 and FBXO7 were responsible for their interaction, plasmids encoding several deletion mutants of both proteins lacking the respective conserved functional and/or structural domain(s) were generated (Fig 2A and 2C). Accordingly, USP7 contains the N-terminal TRAF domain, catalytic domain, and five C-terminal ubiquitin-like (Ubl) domains, primarily comprising two motifs: Ubl-1, -2 (amino acids 560–776) and Ubl-3,

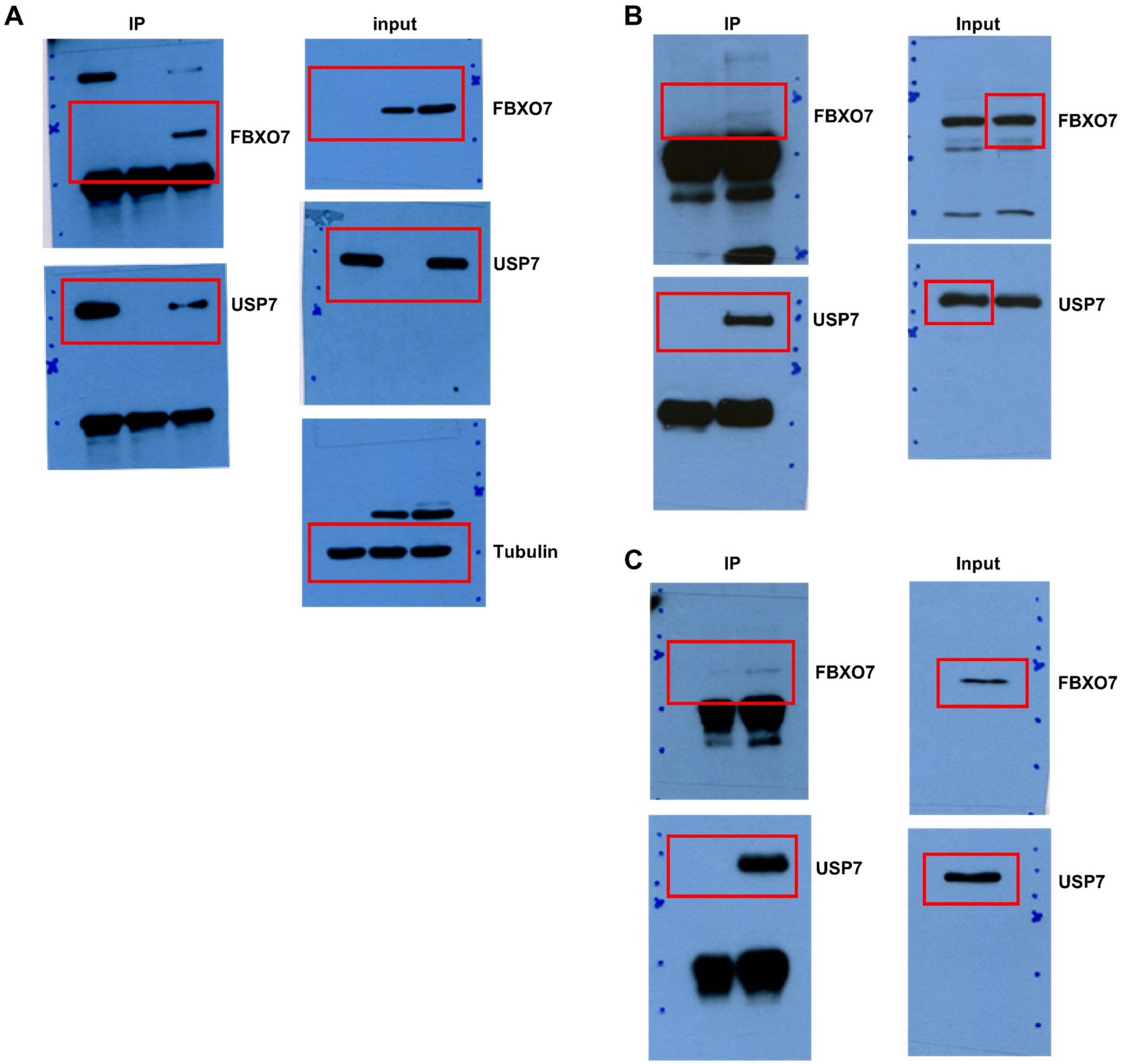

Fig 1. USP7 interacts with FBXO7. (A) After HEK293 cells were transfected for 24 h with plasmids encoding Flag/HA-USP7 and/or Myc-FBXO7, cell lysates were immunoprecipitated using an anti-Flag antibody, followed by immunoblotting with the indicated antibodies. HSP90 served as a loading control. **(B)** HEK293 cells were immunoprecipitated using either anti-USP7 antibody or preimmune IgG (control), followed by immunoblotting with the indicated antibodies. **(C)** SH-SY5Y cell lysates were immunoprecipitated using anti-USP7 IgG, followed by immunoblotting with the indicated antibodies. **(D)** SH-SY5Y cells were fixed, permeabilized, and immunostained. Representative confocal images of cells immunostained for endogenous USP7 (red) and endogenous FBXO7 (green) are shown. Nuclei were counterstained with DAPI (blue). Scale bars denote 10 μm.

https://doi.org/10.1371/journal.pone.0290371.g001

-4, -5 (amino acids 776–1102). FBXO7 has a Ubl domain, FP domain, F-box domain, and proline-rich repeat domain. After expressing each USP7 and FBXO7 mutant in HEK293 cells, their mutual interactions were assessed by co-IP analysis of cell lysates (Fig 2). Wild-type FBXO7 bound to several USP mutants, including USP7-(1–208), USP7-(560–776), and USP7-

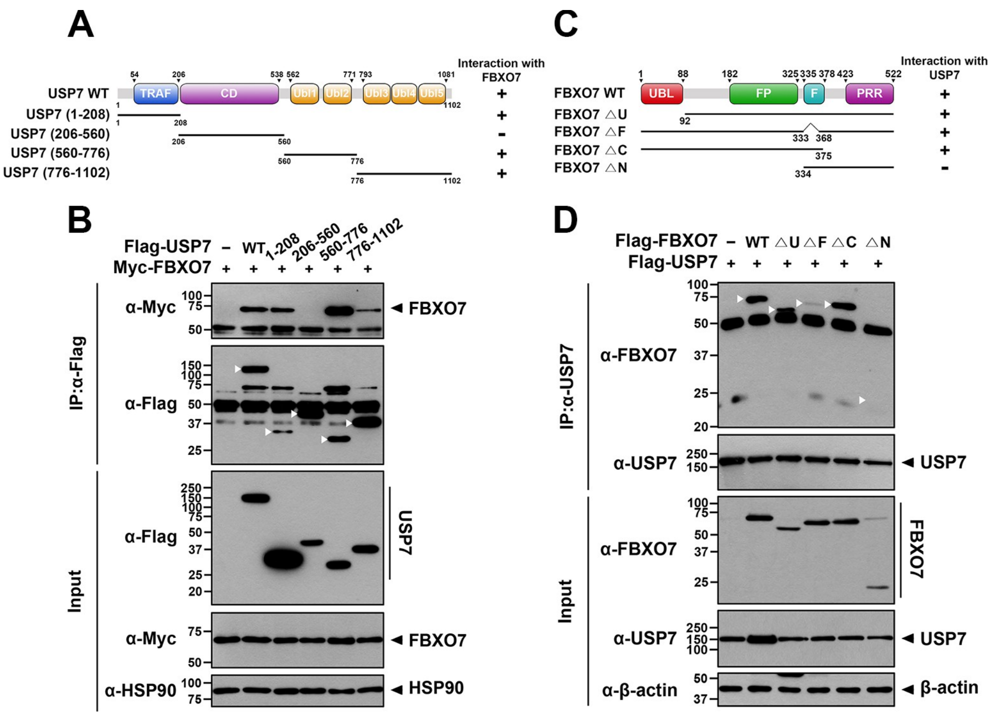

Fig 2. The N- and C-terminal regions of USP7 bind to the central region of FBXO7. **(A)** Schematic diagram of wild-type USP7 (USP7-WT) and its several deletion mutants. The results from co-immunoprecipitation and binding assays between FBXO7 and either USP7-WT or one of its deletion mutants are summarized on the right side. Minus (-) indicates no binding and plus (+) indicates binding. **(B)** Where indicated, HEK293 cells were transfected for 24 h with plasmids encoding Myc-FBXO7, Flag-tagged wild-type USP7, or one of its deletion mutants alone or in combination. Cell lysates were immunoprecipitated using anti-Flag antibody, followed by immunoblotting with the indicated antibodies. **(C)** Schematic diagram of wild-type FBXO7 (FBXO7-WT) and its deletion mutants. The results from co-immunoprecipitation and binding assays between USP7 and either FBXO7-WT or one of its deletion mutants are summarized on the right side. **(D)** HEK293 cells were transfected for 24 h with plasmids encoding Flag-USP7, Flag-tagged wild-type FBXO7, or one of its deletion mutants alone or in combination. Cell lysates were immunoprecipitated using anti-USP7 antibody, followed by immunoblotting with the indicated antibodies. HSP90 served as a loading control.

https://doi.org/10.1371/journal.pone.0290371.g002

(776–1102), and full-length USP7. However, this interaction was not observed in the USP7-(206–560) mutant (Fig 2B). These results suggested that the N-terminal TRAF and C-terminal Ubl domains of USP7 are necessary for the binding of FBXO7. Similarly, additional co-IP analyses revealed that full-length FBXO7 and its deletion mutants, including FBXO7-△U, FBXO7-△F, and FBXO7-△C, bound well to wild-type USP7. However, this interaction was not observed in the FBXO7-△N mutant (Fig 2D). These results suggested that the central region of FBXO7, including the FP domain, is important for its binding of USP7.

Taken together our results indicate that USP7 binds to FBXO7 via the N- and C-terminal regions of USP7 and the central region of FBXO7.

USP7 causes the increase of FBXO7 level

To gain further insight into the functional link between USP7 and FBXO7, we examined whether USP7, as a DUB, affects the level of FBXO7, or vice versa. As shown in Fig 3A and 3B, exogenous and endogenous FBXO7 levels in HEK293 cells greatly increased in the presence of USP7 in a dose-dependent manner. In contrast, small interfering RNA (siRNA)-mediated *USP7* knockdown markedly decreased the endogenous FBXO7 levels (Fig 3C). In addition, endogenous FBXO7 levels were significantly decreased in *USP7*-knockout HeLa cells (Fig 3D).

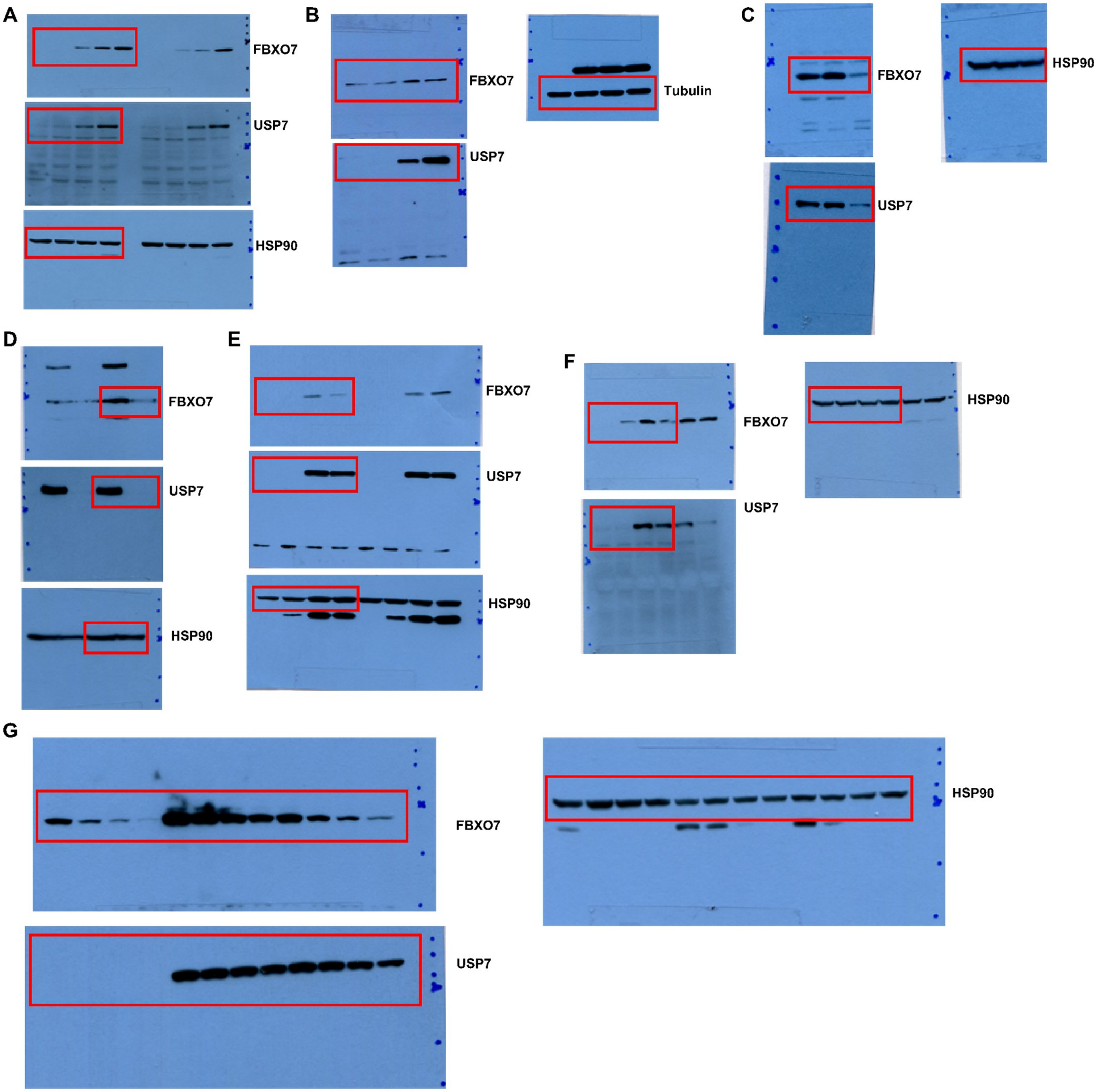

Fig 3. USP7 causes an increase of the FBXO7 level. **(A)** HEK293 cells were transfected for 24 h with a plasmid encoding Myc-FBXO7 alone or together with increasing amounts of plasmid encoding Flag/HA-USP7. Cell lysates were then immunoblotted with anti-Myc or anti-Flag antibodies. The relative FBXO7 level of each sample was quantified and presented as the mean ± SD of three independent experiments (***$p \leq 0.0001$; **$p \leq 0.001$). **(B)** HEK293 cells were transfected for 24 h with increasing amounts of plasmid encoding Flag/HA-USP7. Cell lysates were immunoblotted with anti-FBXO7 or anti-Flag antibodies. **(C)** Where indicated, HEK293 cells were transfected for 48 h with control siRNA or *USP7*-siRNA. Cell lysates were immunoblotted with anti-FBXO7 or anti-USP7 antibodies. **(D)** Immunoblotting analysis of lysates from $USP7^{+/+}$ and $USP7^{-/-}$ HeLa cells was performed using anti-FBXO7 or anti-USP7 antibodies. **(E)** HEK293 cells were transfected for 24 h with plasmids encoding wild type Myc-FBXO7 alone or together with either Flag/HA-USP7-WT or Flag/HA-USP7-CA. Cell lysates were immunoblotted with the indicated antibodies. Relative FBXO7 levels were quantified, and the results are presented as the mean ± SD of three independent experiments (***$p \leq 0.0001$; **$p \leq 0.001$). **(F)** HEK293 cells were transfected for 24 h with plasmids encoding Myc-FBXO7 or/and Flag/HA-USP7. The cells were then treated for an additional 24 h with vehicle (-) or 6.25 μM P5091. **(G)** HEK293 cells were transfected for 24 h with plasmids encoding wild type Myc-FBXO7 alone or together with either Flag/HA-USP7-WT or Flag/HA-USP7-C223A. Cells were then treated for the indicated times with 25 μg/ml cycloheximide, and cell lysates were immunoblotted with the indicated antibodies. Relative FBXO7 levels were quantified and the results are presented as the mean ± SD of three independent experiments (***$p \leq 0.0001$; **$p \leq 0.001$; * $p \leq 0.05$). HSP90 and β-actin served as a loading control.

https://doi.org/10.1371/journal.pone.0290371.g003

To determine whether the catalytic activity of USP7 is required for the upregulation of FBXO7 expression, HEK293 cells were transfected for 24 h with a plasmid encoding Myc-FBXO7 alone or in combination with either Flag/HA-tagged wild-type USP7 (FLAG-HA-USP-WT) or its point-mutant with an alanine (Ala) substation at C223 (Flag/HA-USP7-C223A), which results in a catalytically inactive mutant of USP7. Immunoblot analysis revealed that the USP7-C223A mutant had no effect on FBXO7 levels, in contrast to the effect of USP7-WT (Fig 3E). Furthermore, treatment with the USP7 inhibitor P5091 considerably suppressed the upregulation of FBXO7 (Fig 3F). These results suggested that the catalytic activity of USP7 is critical for the USP7-induced FBXO7 upregulation.

To determine whether USP7 regulates the stability of FBXO7, HEK293 cells were transfected with a plasmid encoding Myc-tagged FBXO7 alone or in combination with either Flag/HA-USP7-WT or Flag/HA-USP7-C223A and the cells were then treated with 25 μM cycloheximide for various durations. Immunoblotting analysis revealed that the half-life of FBXO7 was significantly increased by USP7-WT (Fig 3G). However, this effect was not observed in the presence of the USP7-C223A mutant (Fig 3G). Overall, these results suggested that USP7 increases the stability of FBXO7.

To further determine whether FBXO7 also affects USP7 levels as a mutual regulatory mechanism, HEK293 cells were transfected with increasing amounts of plasmids encoding Myc-FBXO7. Immunoblot analysis of the cell lysates revealed that exogenous and endogenous USP7 levels were not affected by FBXO7 (S2A and S2B Fig).

Overall, our results suggest that FBXO7 acts as a downstream target of USP7, thereby enhancing the half-life of FBXO7.

USP7 promotes the K48-linked deubiquitination of FBXO7

Next we investigated whether USP7 catalyzes the deubiquitination of FBXO7. HEK293 cells were transfected with plasmids encoding HA-ubiquitin, Myc-FBXO7, Flag-USP7-WT, and Flag-USP7-C223S, alone or in combination. Co-IP analysis revealed that co-expression of FBXO7 with wild-type USP7 significantly decreased the level of ubiquitinated FBXO7, whereas this effect was not observed with the catalytic-inactive USP7-C223S mutant (Fig 4A). These results were further confirmed by reciprocal co-IP assay (S3 Fig).

We then examined whether the USP7-mediated deubiquitination of FBXO7 specifically targeted either Lys48- or Lys63-linked ubiquitin chains. HEK293 cells were transfected with plasmids encoding HA-Ub-K48 or HA-Ub-K63 alone, in which six of the seven lysine residues (K6, K11, K27, K29, K33, K48, and K63) of ubiquitin, except K48 or K63, were substituted with alanine, or with either Flag-USP7-WT or Flag-USP-CS. Immunoblotting of cell lysates with an anti-ubiquitin antibody revealed that USP7 promoted the deubiquitination of FBXO7 via the Lys48-ubiquitin linkage destined for degradation by the 26S proteasome pathway, but not via the Lys63-ubiquitin linkage (Fig 4B and 4C). These results suggested that USP7 prevents the proteasomal degradation of FBXO7 through Lys48-linked deubiquitination.

USP7 reduces the TM-induced cytotoxic effect through stabilization of FBXO7

Pharmacological inhibition of USP7 in cancer cells has been shown to strongly increase the accumulation of polyubiquitinated proteins [25]. In addition, USP7 inhibitors induce apoptosis by increasing both oxidative stress and ER stress. Based on these findings, it is highly probable that USP7-mediated stabilization of FBXO7 affects ER stress-induced apoptosis. To explore this possibility, we examined whether the presence of USP7 and/or FBXO7 affects cell viability during the ER stress response. First, $USP7^{+/+}$ or $USP7^{-/-}$ HeLa cells were treated with

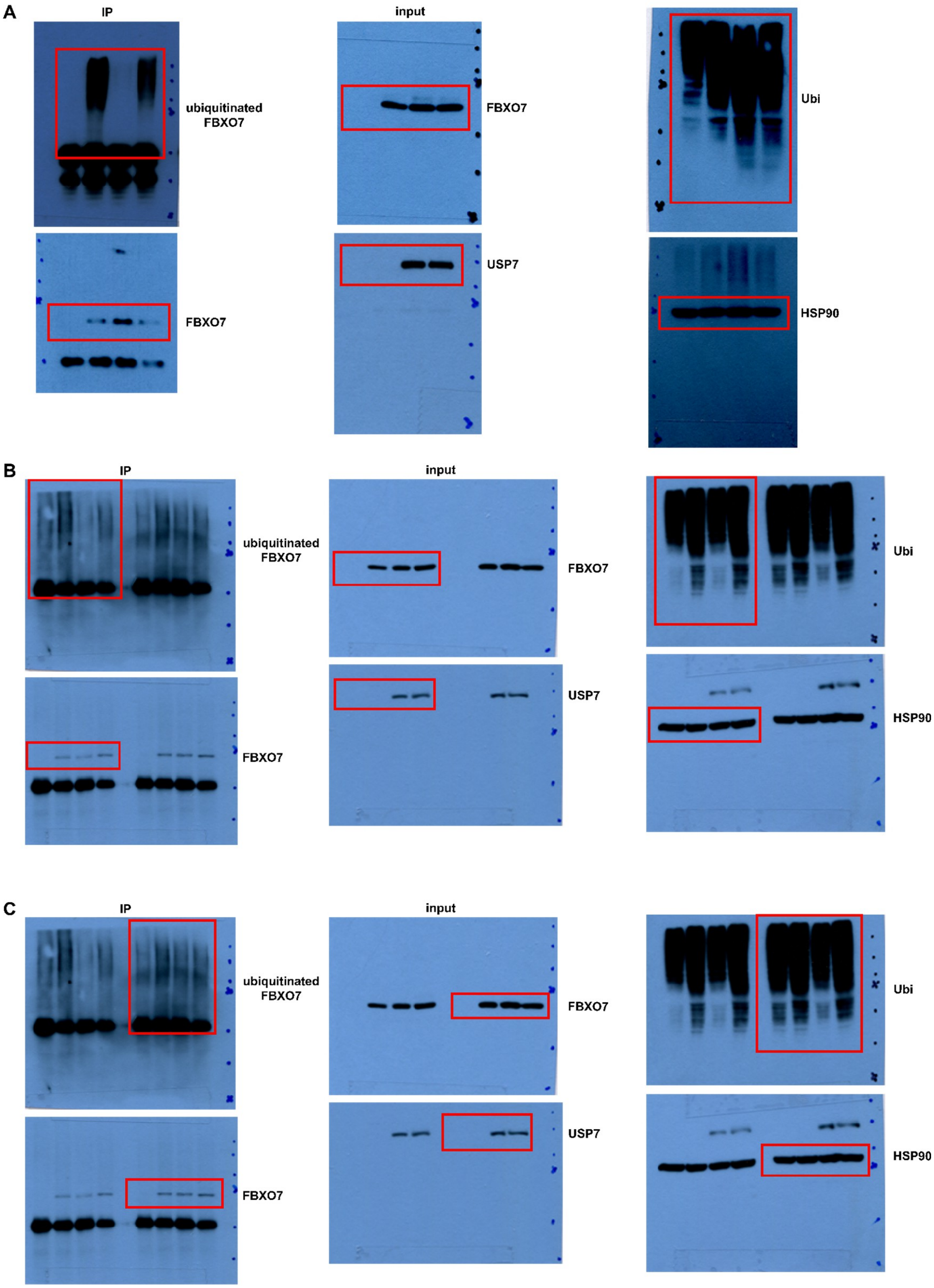

Fig 4. USP7 promotes the K48-linked deubiquitination of FBXO7. (A-C) Where indicated, HEK293 cells were transfected for 24 h with the plasmids encoding HA-Ubiquitin-WT (Ub; A), HA-Ub-K48 in which six Lys residues, except the K48 site, are replaced with alanine (B) or HA-Ub-K63 in which six Lys residues, except the K63 site, are replaced to alanine (C) alone or together with either Flag-USP7-WT or Flag-USP7-CS (A-C), and treated for an additional 6 h with 20 μM MG132. Cell lysates were immunoprecipitated using anti-Myc antibody, followed by immunoblotting with the indicated antibodies. HSP90 served as a loading control.

https://doi.org/10.1371/journal.pone.0290371.g004

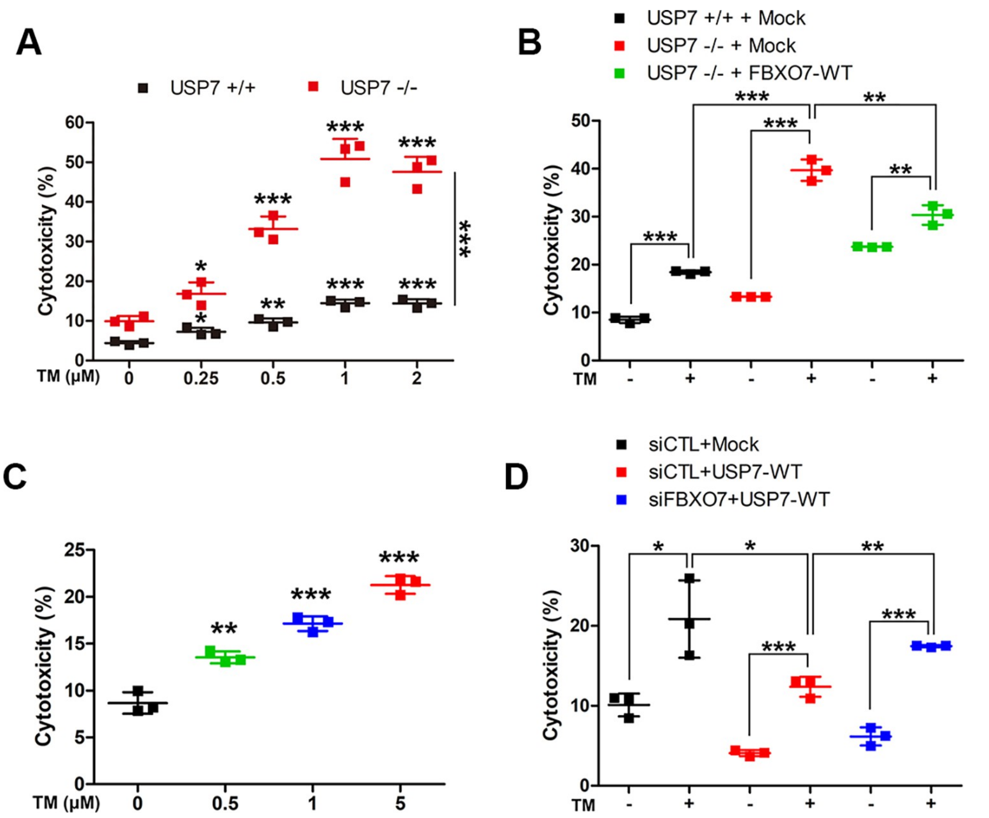

Fig 5. USP7 reduces tunicamycin (TM)-induced cytotoxicity through stabilization of FBXO7. **(A)** $USP7^{+/+}$ and $USP7^{-/-}$ HeLa cells were treated for 24 h with vehicle (-) or the indicated concentrations of TM. Cell toxicity was measured using lactate dehydrogenase (LDH) assays. Data are presented as the mean ± SD of three independent experiments (***$p \leq 0.0001$; **$p \leq 0.001$; *$p \leq 0.05$). **(B)** $USP7^{+/+}$ and $USP7^{-/-}$ HeLa cells were transfected for 24 h with a plasmid encoding Myc-FBXO7, and treated for an additional 24 h with vehicle or TM (1 μM). Data are presented as the mean ± SD of three independent experiments (***$p \leq 0.0001$; **$p \leq 0.001$). **(C)** SH-SY5Y cells were treated for 24 h with vehicle (-) or the indicated concentration of TM. Data are presented as the mean ± SD of three independent experiments (***$p \leq 0.0001$; **$p \leq 0.001$). **(D)** SH-SY5Y cells were transfected for 48 h with control siRNA (CTL), *FBXO7*-siRNA, or a plasmid encoding Flag-USP7, and then treated for an additional 24 h with vehicle or TM (0.5 μM). Data are presented as the mean ± SD of three independent experiments (***$p \leq 0.0001$; **$p \leq 0.001$; *$p \leq 0.05$).

https://doi.org/10.1371/journal.pone.0290371.g005

various concentrations of TM, an inducer of ER stress via inhibition of N-linked glycosylation, and the change in cell viability was measured using a LDH cytotoxicity assay. This assay measures the release of cytoplasmic LDH enzyme as an indicator of plasma membrane damage.

As expected, TM treatment increased cytotoxicity in both $USP7^{+/+}$ and $USP7^{-/-}$ HeLa cells in a dose-dependent manner (Fig 5A). Interestingly, the cytotoxicity to $USP7^{-/-}$ cells was significantly higher than that to $USP7^{+/+}$ cells (Fig 5A). After transfection with a plasmid encoding Myc-FBXO7 for 24 h, followed by treatment with vehicle or 1 μM TM, the $USP7^{-/-}$ cells showed more than 2-fold greater TM-induced cytotoxicity than $USP7^{+/+}$ cells. Moreover, $USP7^{-/-}$ cells transfected with a plasmid encoding Myc-FBXO7 showed a ~10% lower level of TM-induced cytotoxicity compared with that of mock-transfected $USP7^{-/-}$ cells (Fig 5B).

Next, we attempted to determine whether the same effect could be observed in dopaminergic neuroblastoma SH-SY5Y cells. Similar to the results in HEK293 cells, the cytotoxicity of TM to SH-SY5Y cells increased in a dose-dependent manner (Fig 5C). After transfection of SH-SY5Y cells for 48 h with control siRNA, *FBXO7*-siRNA, and/or a plasmid encoding Flag-USP7, followed by treatment with the vehicle or 0.5 μM TM, immunoblotting analysis revealed

that cells expressing Flag-USP7 showed 8% lower levels of TM-induced cytotoxicity compared with that of mock-transfected cells. In addition, the cells expressing Flag-USP7 with *FBXO7* knockdown exhibited a 5% higher level of TM-induced cytotoxicity compared with that of cells expressing Flag-USP7 alone (Fig 5D).

Taken together, these findings suggested that USP7 reduces the cytotoxic effects of TM by stabilizing FBXO7.

USP7 alleviates ER stress-induced apoptosis through stabilization of FBXO7

Next, we examined whether the TM-induced cytotoxicity was due to apoptotic cell death. After USP7$^{+/+}$ and USP7$^{-/-}$ HeLa cells were treated with various concentrations of TM, immunoblot analysis of cell lysates with antiserum against several apoptosis markers confirmed that TM triggered apoptosis. Similar to the cytotoxicity pattern, the levels of cleaved PARP1 and CHOP were much higher in USP7$^{-/-}$ cells than in USP7$^{+/+}$ cells (Fig 6A). We examined whether the same effect could be observed in SH-SY5Y cells. After SH-SY5Y cells were treated with TM alone or in combination with increasing concentrations of the USP7 inhibitor P5091, immunoblot analysis revealed that both of cleaved PARP1 and caspase-3 levels were increased by TM alone, and were further increased by combined treatment of TM and P5091 (S4A Fig). These results suggested that USP7 attenuates ER stress-induced apoptosis.

We further examined whether FBXO7 plays an anti-apoptotic role during TM-induced apoptotic cell death. Similar to the pattern shown in Fig 6A, immunoblot analysis

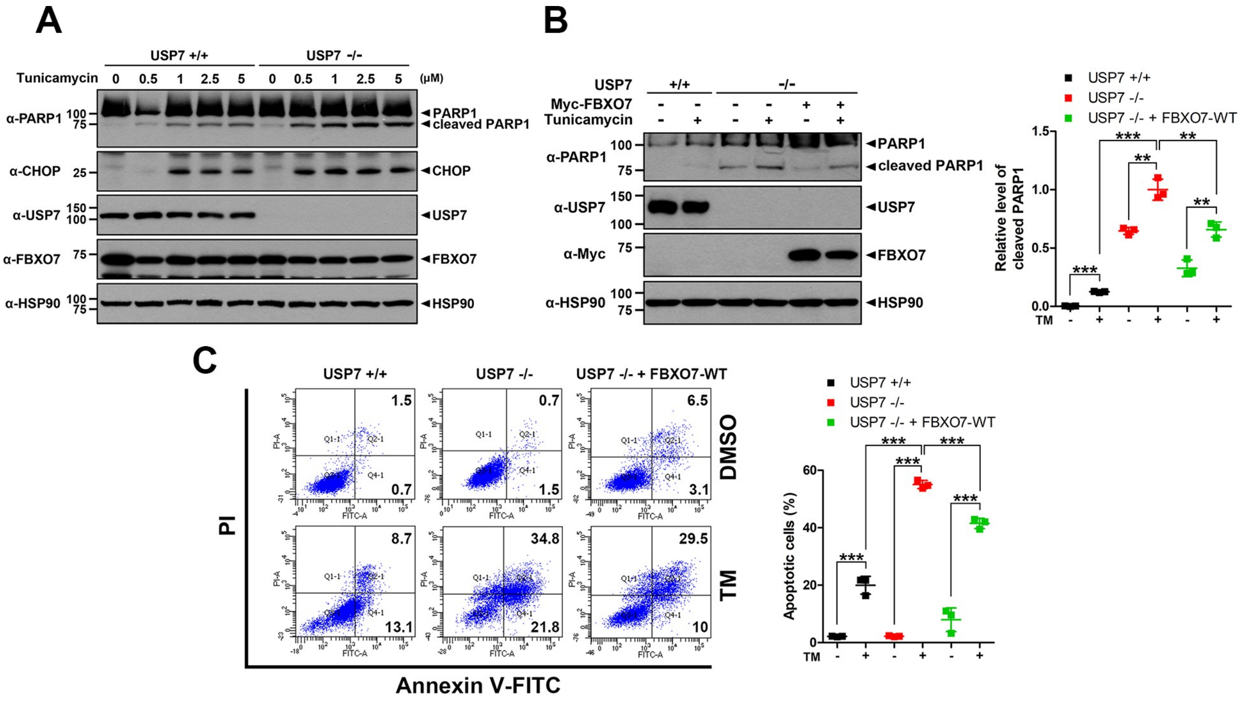

Fig 6. USP7 attenuates ER stress-induced apoptosis through stabilization of FBXO7. **(A)** USP7$^{+/+}$ and USP7$^{-/-}$ HeLa cells were treated for 24 h with vehicle (-) or the indicated concentrations of tunicamycin (TM). Cell lysates were immunoblotted with the indicated antibodies. **(B)** Where indicated, USP7$^{+/+}$ or USP7$^{-/-}$ HeLa cells were transfected for 24 h with a plasmid encoding Myc-FBXO7, and treated for an additional 24 h with vehicle (-) or TM (0.5 μM). Cell lysates were immunoblotted with the indicated antibodies. Data are presented as the mean ± SD of three independent experiments (***$p \leq 0.0001$; **$p \leq 0.001$). HSP90 served as a loading control. **(C)** USP7$^{+/+}$ and USP7$^{-/-}$ HeLa cells were transfected for 24 h with vehicle (dimethyl sulfoxide, DMSO) or TM (1 μM). The extent of apoptotic cell death was detected by flow cytometry. Data are presented as the mean ± SD of three independent experiments (***$p \leq 0.0001$).

https://doi.org/10.1371/journal.pone.0290371.g006

demonstrated that USP7$^{-/-}$ cells displayed 7.8-fold higher levels of cleaved PARP1 under TM treatment than USP7$^{+/+}$ cells (Fig 6B). In addition, when USP7$^{-/-}$ cells were transfected with a plasmid encoding Myc-FBXO7 followed by TM treatment, the cleaved PAPR1 level was decreased by 1.5-fold compared with that of TM-treated and mock-transfected USP7$^{-/-}$ cells (Fig 6B). SH-SY5Y cells were also transfected with a plasmid encoding Myc-FBXO7, followed by co-treatment of TM and P5091, the *USP7*-specific inhibitor. Similar to the pattern shown in Fig 6B, immunoblot analysis demonstrated that SH-SY5Y cells transiently expressing Myc-FBXO7 and treated with TM and P5091 showed a 3.9-fold lower level of cleaved caspase-3 than mock-transfected cells (S4B Fig).

To verify the effect of FBXO7 on TM-induced apoptosis, flow cytometry analysis was performed using Annexin V-FITC and PI staining. As shown in Fig 6C, USP7$^{+/+}$ cells exhibited an 18% higher apoptotic rate with TM treatment compared to untreated cells as a control. After TM treatment, USP7$^{-/-}$ cells showed a 35% increased apoptotic rate compared with that of USP7$^{+/+}$ cells. In addition, USP7$^{-/-}$ HeLa cells expressing Myc-FBXO7 exhibited a 13.6% lower TM-induced apoptotic rate than mock-transfected USP7$^{-/-}$ HeLa cells (Fig 6C).

Overall, these results suggested that USP7 alleviates ER stress-induced apoptosis by stabilizing FBXO7.

USP7 increases the protein stability of three PD-linked mutants as well as wild-type FBXO7

Finally, we investigated the effect of USP7 on three familial PD-associated pathological mutants of FBXO7: two point-mutants of FBXO7 in which the Thr-22 or Arg-378 residues are substituted with Met and Gly, respectively (FBXO7-T22M and FBXO7-R378G), and a nonsense mutant at Arg-498 (FBXO7-R498X) (Fig 7A). HEK293 cells were transfected with a plasmid encoding Flag/HA-USP7, wild-type FBXO7, or one of the FBXO7 mutants alone or in combination. Immunoblot analysis of cell lysates revealed that USP7 increased all three point mutants of FBXO7, similar to the level of wild-type FBXO7 (Fig 7B). However, this effect was not observed in the catalytic inactive USP7-C223S mutant (Fig 7C). These results indicated that USP7 could increase the levels of three PD-associated pathogenic mutants of FBXO7, as well as its wild-type protein in a catalytic-dependent manner.

We further examined the effect of PD-linked mutants on ER stress-induced apoptosis. USP7$^{-/-}$ HeLa cells transfected with the plasmid encoding either Flag-tagged wild-type FBXO7 or one of its PD-linked mutants, and then treated for an additional 24 h with vehicle or TM. Immunoblotting analysis revealed that TM-induced ER stress upregulated the level of cleaved PARP1, which was then decreased in cells expressing FBXO7-R378G, FBXO7-R498X, and wild-type FBXO7 (Fig 7D). However, FBXO7-T22M impaired the ability of FBXO7 to mitigate ER stress-induced apoptosis (Fig 7D). The Ubl domain of FBXO7 is important for interaction with parkin, which recruits parkin into damaged mitochondria to initiate mitophagy. FBXO7-T22M mutation within the Ubl domain interferes with its interaction with parkin [26]. Our results suggested that the interaction between FBXO7 and parkin appears to be essential for mitigating ER stress-induced apoptosis.

Taken together, these findings highlight the importance of USP7 in promoting the interaction between FBXO7 and parkin, thereby contributing to the regulation of cellular responses to ER stress.

Discussion

ER stress is considered to mainly contribute to the degeneration of dopaminergic neurons, which are particularly sensitive to unfolded, misfolded, and aggregated proteins [27].

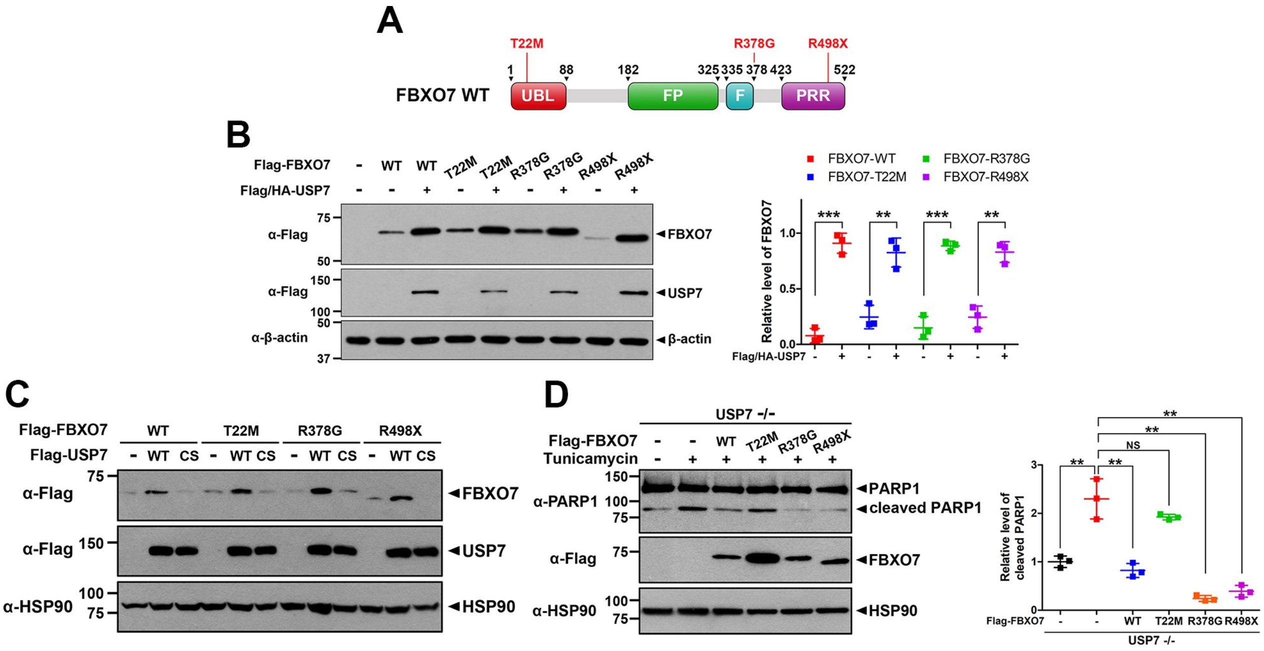

Fig 7. USP7 enhances the levels of three Parkinson's disease (PD)-linked FBXO7 mutants as well as wild-type FBXO7. (**A**) Schematic diagram of wild-type FBXO7 and its three PD-linked mutants (FBXO7-T22M, FBXO7-R378G, and FBXO7-R498X). (**B**) HEK293 cells were transfected for 24 h with plasmids encoding Flag/HA-USP7, Flag-FBXO7-WT, Flag-FBXO7-T22M, Flag-FBXO7-R378G, or Flag-FBXO7-R498X alone or in combination. Cell lysates were immunoblotted with the indicated antibodies. The relative amount of FBXO7 in each sample was quantified and data are presented as the mean ± SD of three independent experiments (****p* ≤ 0.0001; ***p* ≤ 0.001). (**C**) HEK293 cells were transfected for 24 h with plasmids encoding Flag-USP7-WT, Flag-USP7-C223S, Flag-FBXO7-WT, Flag-FBXO7-T22M, Flag-FBXO7-R378G, or Flag-FBXO7-R498X alone or in combination. Cell lysates were immunoblotted with the indicated antibodies. (**D**) $USP7^{-/-}$ HeLa cells were transfected for 24 h with the plasmids encoding either Flag-tagged wild-type FBXO7 or one of its PD-linked mutants, and then treated for an additional 24 h with vehicle (-) or TM (0.5 μM). Cell lysates were immunoblotted with the indicated antibodies. Data are presented as the mean ± SD of three independent experiments (***p* ≤ 0.001; NS, not significant). β-actin and HSP90 served as a loading control.

https://doi.org/10.1371/journal.pone.0290371.g007

Accordingly, many recent studies have indicated a correlation between ER stress and the pathogenesis of PD. For example, ATF4-upregulated parkin suppresses ER stress and cell death via E3 ubiquitin ligase activity [28, 29]. Furthermore, TM- or thapsigargin-induced ER stress inhibits the loss of parkin activity and promotes its recruitment to the mitochondria, thereby activating mitophagy during reperfusion after ischemia [30]. Another previous study revealed that FBXO7 interacts with parkin and recruits parkin into impaired mitochondria, which facilitates mitophagy to protect cells [26]. These hypotheses were further supported by our finding that the USP7-mediated stabilization of FBXO7 inhibited cell death under ER stress. Moreover, treatment with USP7 inhibitors has been reported to cause the accumulation of polyubiquitinated proteins, leading to ER stress induction in cancer cells. Consequently, induction of ER stress by USP7 inhibitors induces oxidative stress and cancer cell apoptosis [25]. In the present study, we also found that both *USP7* knockout in HeLa cells and treatment with USP7 inhibitors in SH-SY5Y cells increased ER stress-induced cytotoxicity and apoptosis. In addition, this induction, via the blockade of USP7 activity, was rescued by FBXO7 overexpression. Interestingly, the pathogenic PD-linked FBXO7-T22M mutant, which impairs the interaction with parkin, does not rescue the ER stress-induced apoptosis. These results suggest that USP7-upregulated FBXO7 interacts with parkin, which is necessary for the protective mechanism against ER stress-induced apoptosis. In addition, the altered FBXO7-parkin binding resulting from the T22M mutation may hold significant implications for familial PD cases associated with the variant form of FBXO7, emphasizing its close association with the occurrence of the disease.

The ubiquitin-proteasome system (UPS) is one of the two major protein degradation machineries in eukaryotic cells [31]. Recent studies have shown that several E3 ligases and DUBs, two important components of the UPS, precisely control the ER stress response through the ubiquitination or deubiquitination of target proteins [16]. For example, the mitochondrial E3 ubiquitin ligase MITOL ubiquitinates the mitochondrial fission factor Drp1, affecting mitochondrial morphology and dynamics [32]. MITOL also ubiquitinates the ER transmembrane protein IRE1α and inhibit its activity, thereby limiting ER stress-dependent apoptosis [33]. Moreover, the ER-located RING finger E3 ligase RNF186 was not only found to be upregulated under ER stress but also participated in the regulation of ER stress-mediated apoptosis [34]. The pro-apoptotic protein BNip1 is a substrate of RNF186, which interacts with and colocalizes in the ER. Overexpression of RNF186 in mouse primary hepatocytes also promotes the expression of inflammatory factors, including tumor necrosis factor-α, interleukin-6, and monocyte chemoattractant protein-1, in the ER stress pathway [35]. These results indicate that RNF186 plays a crucial role in apoptosis and the inflammatory response under ER stress. The ubiquitin ligase GP78 ubiquitinates Ubl4A, a component of the ERAD pathway [36]. Interestingly, USP13 counteracts the GP78-mediated ubiquitination of Ubl4A through the deubiquitination and stabilization of Ubl4A, thus playing a positive role in the degradation of misfolded proteins [36]. Finally, ORP8 is an ER-anchored protein implicated in lipid transfer at the contact sites between the ER and other membranes. Recently, it was reported that the interaction between USP5 and ORP8 can induce ER stress [37]. USP5 promotes the accumulation of ORP8 and disturbance of phospholipid transport through ORP8 deubiquitination. USP5-mediated deubiquitination of ORP8 indirectly aggravates ER stress in colon cancer cells and induces apoptosis [37]. In addition to these cases, the present study provides another example, identifying that USP7 and FBXO7 act as novel DUB and E3 ligases involved in the ER stress response. Moreover, our data indicate that the pro-survival effect of USP7 could be exerted through its catalytic activity in response to ER stress.

Similar to the current finding of the USP-mediated modulation of FBXO7 stability, and its proposed link to PD pathology, there have been many reports demonstrating the association between DUBs and two PD-linked genes, *PINK1* and parkin. The mitochondrial kinase PINK1 and the ubiquitin ligase parkin degrade damaged mitochondria via mitophagy. In addition to toxic aggregates of α-synuclein in neurons, mitochondrial dysfunction contributes to PD. Mitophagy is a subtype of macroautophagy that selectively targets defective mitochondria to the lysosome for degradation [38]. Durcan et al. demonstrated that USP8 plays a direct role in deubiquitinating parkin and preventing its auto-ubiquitination [39]. RNA interference-mediated knockdown of *USP8* in different cell lines caused a delay in the recruitment of parkin to depolarized mitochondria. In addition, it led to the accumulation of ubiquitinated parkin, which remained longer in the damaged mitochondria and delayed their clearance by mitophagy. Three additional DUBs were linked to parkin and parkin-mediated mitophagy. USP15 and two mitochondrial USPs, USP30 and USP35, inhibit parkin-mediated ubiquitination of mitochondrial proteins, thereby mitigating the clearance of depolarized mitochondria [40, 41]. Recently, mitochondrial USP33 has the ability to deubiquitinate parkin by removing various types of ubiquitin conjugates (K6-, K11-, K48-, and K63-linked) from parkin [42]. *USP33*-knockdown increased the stability of parkin and its translocation to depolarized mitochondria, which enhanced mitophagy and protection to human neuroblastoma cells against apoptotic cell death induced by the neurotoxin 1-methyl-4-phenyl-1,2,3,6-tetrahydropyridine. Collectively, previous findings and those of the present study support the concept that protein ubiquitination and ubiquitin-dependent proteasomal degradation are balanced by deubiquitination, and defective control during these events somehow contributes to the pathogenesis of PD.

FBXO7 exerts both protective and toxic effects. For example, we previously reported that FBXO7 triggers neuronal cell death in response to oxidative stress through degradation of cytoprotective FOXO4 and SIRT7 [10, 11]. Moreover, FBXO7 promotes the ubiquitination and proteasomal degradation of cytoprotective PINK1 in BEAS-B2 cells [43]. In contrast, mild and transient stress can induce FBXO7 expression, facilitating neuroprotective mitophagy and protecting cells [44]. In addition, FBXO7 differentially affects the stability of PINK1 depending on the cell types [45]. For instance, the presence of wild-type FBXO7 or its familial PD-linked mutants led to the accumulation of PINK1 upon carbonyl cyanide m-chlorophenyl hydrazone (CCCP) treatment in HEK293, HeLa, and SH-SY5Y cells. FBXO7 acts as a scaffold protein with chaperone activity. FBXO7 directly interacts with Bag2, which plays an important role in stabilizing PINK1 by inhibiting its ubiquitination. Therefore, the accumulation of PINK1 is dependent on the synergic chaperone activity of FBXO7 and Bag2. These results suggested that FBXO7 and PINK1 play reciprocal roles in regulating protein levels [45]. FBXO7 levels were found to be decreased in the substantia nigra pars compacta from *Pink1*-knockout mice. Consequently, PINK1 kinase activity may play a crucial role in regulating FBXO7 protein levels and FBXO7-mediated mitophagy [45]. The current study provides evidence that FBXO7 displays a cytoprotective action. We found that USP7-mediated FBXO7 stabilization alleviated ER stress-induced cytotoxicity and apoptosis in SH-SY5Y and *USP7*-knockout HeLa cells. These results further indicated that USP7 increased the protein level of FBXO7 with scaffold activity to restore ER homeostasis in response to ER stress.

In conclusion, we propose a new regulatory pathway for the action of FBXO7. The current findings also suggest that the USP7-mediated stabilization of FBXO7 plays a role in ER stress-induced cell death and PD pathogenesis.

Supporting information

S1 Fig. USP7 indirectly binds to FBXO7.
(PDF)

S2 Fig. FBXO7 does not affect the USP7 level.
(PDF)

S3 Fig. USP7 deubiquitinates the FBXO7.
(PDF)

S4 Fig. USP7 attenuates ER stress-induced apoptosis in SH-SY5Y cells.
(PDF)

S1 Raw image.
(PDF)

S2 Raw image.
(PDF)

S3 Raw image.
(PDF)

S4 Raw image.
(PDF)

Acknowledgments

We thank H.J. Kuiken, J.H. Kim and K.H. Baek for providing plasmids and S. Ramakrishna for providing *USP7*-knockout HeLa cell lines.

Author Contributions

Conceptualization: Su Hyoun Lee, Kwang Chul Chung.

Data curation: Su Hyoun Lee, Kwang Chul Chung.

Formal analysis: Su Hyoun Lee, Kwang Chul Chung.

Funding acquisition: Kwang Chul Chung.

Investigation: Su Hyoun Lee, Kwang Chul Chung.

Methodology: Su Hyoun Lee.

Project administration: Kwang Chul Chung.

Resources: Kwang Chul Chung.

Supervision: Kwang Chul Chung.

Validation: Su Hyoun Lee, Kwang Chul Chung.

Writing – original draft: Su Hyoun Lee, Kwang Chul Chung.

Writing – review & editing: Kwang Chul Chung.

References

1. Samii A Nutt JG, Ransom BR. Parkinson's disease. Lancet. 2004; 29(363):1783–93. https://doi.org/10.1016/S0140-6736(04)16305-8 PMID: 15172778
2. Houlden H, Singleton AB. The genetics and neuropathology of Parkinson's disease. Acta. Neuropathol. 2012; 124(3):325–38. https://doi.org/10.1007/s00401-012-1013-5 PMID: 22806825
3. Li W, Fu Y, Halliday GM, Sue CM. PARK genes link mitochondrial dysfunction and alpha-synuclein pathology in sporadic Parkinson's disease. Front. Cell. Dev. Biol. 2021; 9:612476. https://doi.org/10.3389/fcell.2021.612476 PMID: 34295884
4. Nelson D, Randle SJ, Laman H. Beyond ubiquitination: The atypical functions of Fbxo7 and other F-box proteins. Open Biol. 2013; 3(10):130131. https://doi.org/10.1098/rsob.130131 PMID: 24107298
5. Hsu JM, Lee YC, Yu CT, Huang CY. Fbx7 functions in the SCF complex regulating Cdk1-cyclin B-phosphorylated hepatoma up-regulated protein (HURP) proteolysis by a proline-rich region. J. Biol. Chem. 2004; 279(31):32592–602. https://doi.org/10.1074/jbc.M404950200 PMID: 15145941
6. Chang YF, Cheng CM, Chang LK, Jong YJ, You CY. The F-box protein Fbxo7 interacts with human inhibitor of apoptosis protein cIAP1 and promotes cIAP1 ubiquitination. Biochem. Biophys. Res. Commun. 2006; 342(4):1002–16. https://doi.org/10.1016/j.bbrc.2006.02.061 PMID: 16510124
7. Kang J, Chung KC. The F-box protein FBXO7 positively regulates bone morphogenetic protein-mediated signaling through Lys-63-specific ubiquitination of neurotrophin receptor-interacting MAGE (NRAGE). Cell. Mol. Life Sci. 2015; 72(1):181–95. https://doi.org/10.1007/s00018-014-1665-5 PMID: 24947323
8. Vingill S, Brockelt D, Lancelin C, Tatenhorst L, Dontcheva G, Preisinger C, et al. Loss of FBXO7 (PAKR15) results in reduced proteasome activity and models a parkinsonism-like phenotype in mice. EMBO J. 2016; 35(18):2008–25. https://doi.org/10.15252/embj.201593585 PMID: 27497298
9. Teixeira FR, Randel SJ, Patel SP, Mevissen TE, Zenkeviciute G, Koide T, et al. Gsk3β and Tomm20 are substrates of the SCFFbxo7/PARK15 ubiquitin ligase associated with Parkinson's disease. Biochem. J. 2016; 473(20):3563–3580. https://doi.org/10.1042/BCJ20160387 PMID: 27503909
10. Lee SH, Lee YJ, Jung S, Chung KC. E3 ligase adaptor FBXO7 contributes to ubiquitination and proteasomal degradation of SIRT7 and promotes cell death in response to hydrogen peroxide. J. Biol. Chem. 2023; 299(3):102909. https://doi.org/10.1016/j.jbc.2023.102909 PMID: 36646384

11. Lee SH, Jung S, Lee YJ, Hyun M, Chung KC. FBXO7 triggers caspase 8-mediated proteolysis of the transcription factor FOXO4 and exacerbates neuronal cytotoxicity. J. Biol. Chem. 2021; 297 (6):101426. https://doi.org/10.1016/j.jbc.2021.101426 PMID: 34800438

12. Mao J, Hu Y, Ruan L, Ji Y, Lou Z. Role of endoplasmic reticulum stress in depression. Mol. Med. Rep. 2019; 20(6):4774–4780. https://doi.org/10.3892/mmr.2019.10789 PMID: 31702816

13. Tabas I, Ron D. Integrating the mechanisms of apoptosis induced by endoplasmic reticulum stress. Nat. Cell Biol. 2011; 13(3):184–190. https://doi.org/10.1038/ncb0311-184 PMID: 21364565

14. Wiseman RL, Mesgarzadeh JS, Hendershot LM. Reshaping endoplasmic reticulum quality control through the unfolded protein response. Mol. Cell. 2022; 82(8):1477–1491. https://doi.org/10.1016/j.molcel.2022.03.025 PMID: 35452616

15. Hiramatsu N, Chiang WC, Kurt TD, Sigurdson CJ, Lin JH. Multiple mechanisms of unfolded protein response-induced cell death. Am. J. Pathol. 2015; 185(7):1800–8. https://doi.org/10.1016/j.ajpath.2015.03.009 PMID: 25956028

16. Qu J, Zou T, Lin Z. The roles of the ubiquitin-proteasome system in the endoplasmic reticulum stress pathway. Int J Mol Sci. 2021; 22(4):1526. https://doi.org/10.3390/ijms22041526 PMID: 33546413

17. Merighi A, Lossi L. Endoplasmic reticulum stress signaling and neuronal cell death. Int. J. Mol. Sci. 2022; 23(23):15186. https://doi.org/10.3390/ijms232315186 PMID: 36499512

18. Sano R, Reed JC. ER stress-induced cell death mechanisms. Biochim. Biophys. Acta. 2013; 1833 (12):3460–3470. https://doi.org/10.1016/j.bbamcr.2013.06.028 PMID: 23850759

19. Kim S, Kim DK, Jeong S, Lee J. The Common cellular events in the neurodegenerative diseases and the associated role of endoplasmic reticulum stress. Int. J. Mol. Sci. 2022; 23(11):5894. https://doi.org/10.3390/ijms23115894 PMID: 35682574

20. Nininahazwe L, Liu B, He C, Zhang H, Chen ZS. The emerging nature of ubiquitin-specific protease 7 (USP7): a new target in cancer therapy. Drug Discov. Today. 2021; 26(2):490–502. https://doi.org/10.1016/j.drudis.2020.10.028 PMID: 33157193

21. Harakandi C, Nininahazwe L, Xu H, Liu B, He C, Zhen YC, et al. Recent advances on the intervention sites targeting USP7-MDM2-p53 in cancer therapy. Bioorg. Chem. 2021; 116:105273. https://doi.org/10.1016/j.bioorg.2021.105273 PMID: 34474304

22. Al-Eidan A, Wang Y, Skipp P, Ewing RM. The USP7 protein interaction network and its roles in tumorigenesis. Genes Dis. 2020; 9(1), 41–50. https://doi.org/10.1016/j.gendis.2020.10.004 PMID: 35005106

23. Jiang L, Xiong J, Zhan J, Yuan F, Tang M, Zhang C, et al. Ubiquitin-specific peptidase 7 (USP7)-mediated deubiquitination of the histone deacetylase SIRT7 regulates gluconeogenesis. J. Biol. Chem. 2017; 292(32):13296–13311 https://doi.org/10.1074/jbc.M117.780130 PMID: 28655758

24. van der Horst A, de Vries-Smits AM, Brenkman AB, van Triest MH, van den Broek N, Colland F, et al. FOXO4 transcriptional activity is regulated by monoubiquitination and USP7/HAUSP. Nat. Cell Biol. 2006; 8(10):1064–73. https://doi.org/10.1038/ncb1469 PMID: 16964248

25. Lee G, Oh TI, Um KB, Yoon H, Soh J, Kim BM, et al. Small-molecule inhibitors of USP7 induce apoptosis through oxidative and endoplasmic reticulum stress in cancer cells. Biochem. Biophys. Res. Commun. 2016; 470(1):181–186. https://doi.org/10.1016/j.bbrc.2016.01.021 PMID: 26768359

26. Burchell VS, Nelson DE, Sanchez-Martinez A, Delgado-Camprubi M, Ivatt RM, Pogson JH, et al. The Parkinson's disease-linked proteins Fbxo7 and Parkin interact to mediate mitophagy. Nat. Neurosci. 2013; 16(9):1257–65. https://doi.org/10.1038/nn.3489 PMID: 23933751

27. Ren H, Zhai W, Lu Xiaojun, Wang G. The cross-links of endoplasmic reticulum stress, autophagy, and neurodegeneration in Parkinson's disease. Front. Aging Neurosci. 2021; 13:691881. https://doi.org/10.3389/fnagi.2021.691881 PMID: 34168552

28. Imai Y, Soda M, Takahashi R. Parkin suppresses unfolded protein stress-induced cell death through its E3 ubiquitin-protein ligase activity. J. Biol. Chem. 2000; 275(46):35661–4. https://doi.org/10.1074/jbc.C000447200 PMID: 10973942

29. Bouman L, Schlierf A, Lutz AK, Shan J, Deinlein A, Kast J. Parkin is transcriptionally regulated by ATF4: evidence for an interconnection between mitochondrial stress and ER stress. Cell Death Differ. 2011; 18(5):769–82. https://doi.org/10.1038/cdd.2010.142 PMID: 21113145

30. Zhang X, Yuan Y, Jiang L, Zhang J, Gao J, Shen Z. Endoplasmic reticulum stress induced by tunicamycin and thapsigargin protects against transient ischemic brain injury: Involvement of PARK2-dependent mitophagy. Autophagy. 2014; 10(10):1801–13. https://doi.org/10.4161/auto.32136 PMID: 25126734

31. Dikic I. Proteasomal and autophagic degradation systems. Annu. Rev. Biochem. 2017; 86:193–224. https://doi.org/10.1146/annurev-biochem-061516-044908 PMID: 28460188

32. Nagashima S, Tokuyama T, Yonashiro R, Inatome R, Yanagi S. Roles of mitochondrial ubiquitin ligase MITOL/MARCH5 in mitochondrial dynamics and disease. J. Biochem. 2014; 155(5):273–9. https://doi.org/10.1093/jb/mvu016 PMID: 24616159

33. Takeda K, Nagashima S, Shiiba I, Uda A, Tokuyama T, Ito N, et al. MITOL prevents ER stress-induced apoptosis by IRE1alpha ubiquitylation at ER-mitochondria contact sites. EMBO J. 2019; 38(15): e100999. https://doi.org/10.15252/embj.2018100999 PMID: 31368599

34. Wang P, Wu Y, Li Y, Zheng J, Tang J. A novel RING finger E3 ligase RNF186 regulate ER stress-mediated apoptosis through interaction with BNip1. Cell signal. 2013; 25(11):2320–33. https://doi.org/10.1016/j.cellsig.2013.07.016 PMID: 23896122

35. Tong X, Zhang Q, Wang L, Ji Y, Zhang L, Xie L, et al. RNF186 impairs insulin sensitivity by inducing ER stress in mouse primary hepatocytes. Cell. Signal. 2018; 52:155–162. https://doi.org/10.1016/j.cellsig.2018.09.008 PMID: 30223017

36. Liu Y, Soetandyo N, Lee JC, Liu L, Xu Y, Clemons WM, et al. USP13 antagonizes gp78 to maintain functionality of a chaperone in ER-associated degradation. eLife. 2014; 3:e01369. https://doi.org/10.7554/eLife.01369 PMID: 24424410

37. Zhang Z, Gao W, Zhou L, Chen Y, Qin S, Zhang L, et al. Repurposing Brigatinib for the Treatment of Colorectal Cancer Based on Inhibition of ER-phagy. Theranostics. 2019; 9(17):4878–4892. https://doi.org/10.7150/thno.36254 PMID: 31410188

38. Um JH, Yun JH. Emerging role of mitophagy in human diseases and physiology. BMB Rep. 2017; 50 (6):299–307. https://doi.org/10.5483/bmbrep.2017.50.6.056 PMID: 28366191

39. Durcan TM, Tang MY, Pérusse JR, Dashti EA, Aguileta MA, McLelland GL, et al. USP8 regulates mitophagy by removing K6-linked ubiquitin conjugates from parkin. EMBO J. 2014; 33(21):2473–91. https://doi.org/10.15252/embj.201489729 PMID: 25216678

40. Bingol B, Tea JS, Phu L, Reichelt M, Bakalarski CE, Song Q, et al. The mitochondrial deubiquitinase USP30 opposes Parkin-mediated mitophagy. Nature. 2014; 510(7505):370–5. https://doi.org/10.1038/nature13418 PMID: 24896179

41. Cornelissen T, Haddad D, Wauters F, Van Humbeeck C, Mandemakers W, Koentjoro B, et al. The deubiquitinase USP15 antagonizes Parkin-mediated mitochondrial ubiquitination and mitophagy. Hum. Mol. Genet. 2014; 23(19):5227–42. https://doi.org/10.1093/hmg/ddu244 PMID: 24852371

42. Niu K, Fang H, Chen Z, Zhu Y, Tan Q, Wei D, et al. USP33 deubiquitinates PRKN/Parkin and antagonizes its role in mitophagy. Autophagy. 2020; 16(4):724–734. https://doi.org/10.1080/15548627.2019.1656957 PMID: 31432739

43. Liu Y, Bear TB, Verma M, Wang KZ, Otero PA, McKelvey A, et al. Chemical inhibition of FBXO7 reduces inflammation and confers neuroprotection by stabilizing the mitochondrial kinase PINK1. JCI Insight. 2020; 5(11):e131834. https://doi.org/10.1172/jci.insight.131834 PMID: 32493843

44. Zhou ZD, Xie SP, Sathiyamoorthy S, Saw WT, Sing TY, Ng SH, et al. F-box protein 7 mutations promote protein aggregation in mitochondria and inhibit mitophagy. Hum. Mol. Genet. 2015; 24(22):6314–30. https://doi.org/10.1093/hmg/ddv340 PMID: 26310625

45. Huang T, Fang L, He R, Weng H, Chen X, Ye Q, et al. Fbxo7 and Pink1 play a reciprocal role in regulating their protein levels. Aging (Albany NY). 2020; 13(1):77–88. https://doi.org/10.18632/aging.202236 PMID: 33291077
